# Supplementary material for: CITED2 coordinates key hematopoietic regulatory pathways to maintain the HSC pool in both steady-state hematopoiesis and transplantation
Source: Stem Cell Reports. 2021 Oct 28;16(11):2784–97. doi: 10.1016/j.stemcr.2021.10.001 (PMC8581166; doi:10.1016/j.stemcr.2021.10.001)
Supplement: Document S1. Supplemental experimental procedures, Figures S1–S4 [file mmc1.pdf]

Stem Cell Reports, Volume *16*

Supplemental Information

CITED2 coordinates key hematopoietic regulatory pathways to maintain the HSC pool in both steady-state hematopoiesis and transplantation

Hannah Lawson, Louie N. van de Lagemaat, Melania Barile, Andrea Tavosanis, Jozef Durko, Arnaud Villacreces, Aarushi Bellani, Christopher Mapperley, Elise Georges, Catarina Martins-Costa, Catarina Sepulveda, Lewis Allen, Joana Campos, Kirsteen J. Campbell, Dónal O'Carroll, Berthold Göttgens, Suzanne Cory, Neil P. Rodrigues, Amelie V. Guitart, and Kamil R. Kranc

Supplemental Figures

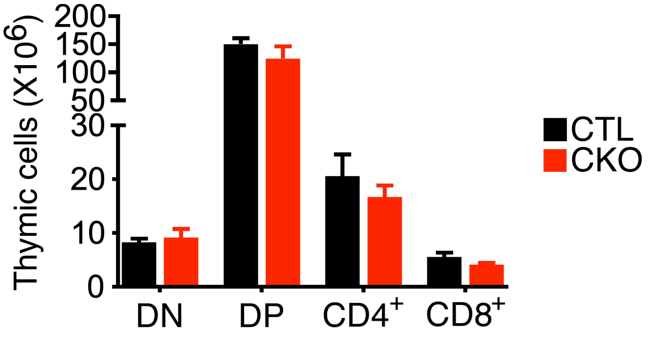

Figure S1. Haematopoiesis-specific *Cited2* deletion has no impact on T cell development. Total number of double-negative (DN; $CD4^-CD8^-$), double-positive (DP; $CD4^+CD8^+$), $CD4^+$ and $CD8^+$ T cells in the thymi. Data are mean ± SEM (n=6-9).

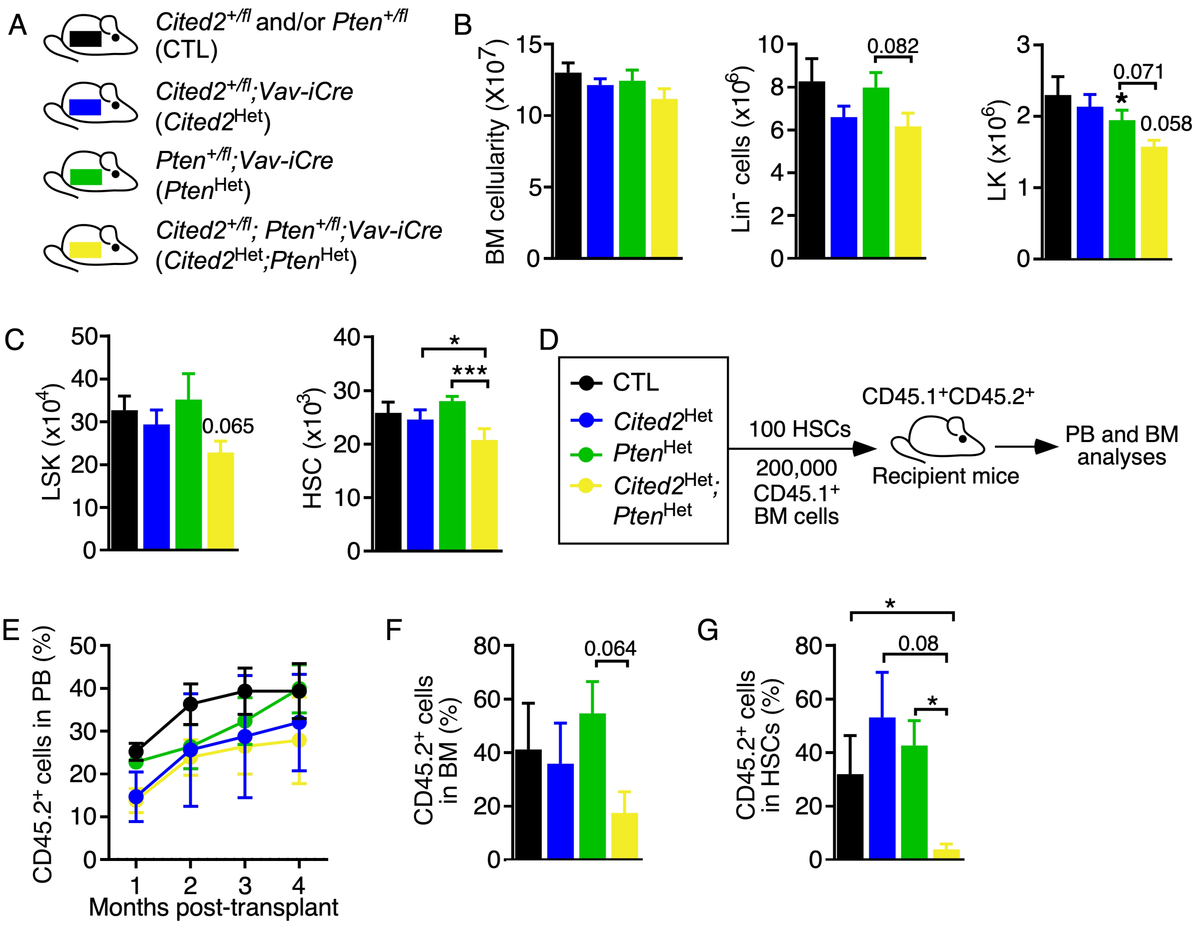

Figure S2. Deletion of one allele of *Pten* impairs maintenance of HSCs lacking one allele of *Cited2*. (**A**) Schematic representation of experimental mouse cohorts; *Cited2*$^{+/fl}$ or *Pten*$^{+/fl}$ (CTL), *Cited2*$^{+/fl}$*;Vav-iCre* (*Cited2*^{HET}), *Pten*$^{+/fl}$*;Vav-iCre* (*Pten*^{HET}) and *Cited2*$^{+/fl}$;*Pten*$^{+/fl}$*;Vav-iCre* (*Cited2*^{HET};*Pten*^{HET}). (**B**) BM cellularity and total numbers of Lin$^-$ cells, LK cells in BM of 8 to 10-week-old mice (n=6-8). Data are mean ± SEM. (**C**) Total numbers of LSK cells and HSCs in BM of 8 to 10-week-old mice (n=6-8). Data are mean ± SEM.(**D**) Transplantation assay: 100 HSCs from CTL, *Cited2*^{HET}, (*Pten*^{HET}) and *Cited2*^{HET}*;Pten*^{HET} were transplanted into lethally irradiated recipients together with 2x10^5 support CD45.1$^+$ total BM cells. (**E**) Percentage of donor-derived CD45.2$^+$ cells in PB following transplantation (n=4 recipients per donor; number of donors: CTL n=3, *Cited2*^{HET} n=3, *Pten*^{HET} n=3, *Cited2*^{HET};*Pten*^{HET} n=3). Data are mean ± SEM. (**F**) Percentage of donor-derived CD45.2$^+$ cells in total BM compartment of the recipient mice 16 weeks after transplantation. Data are mean ± SEM. (**G**) Percentage of donor-derived CD45.2$^+$ cells in the BM HSC compartment. Data are mean ± SEM. *, P<0.05; ***, P<0.001.

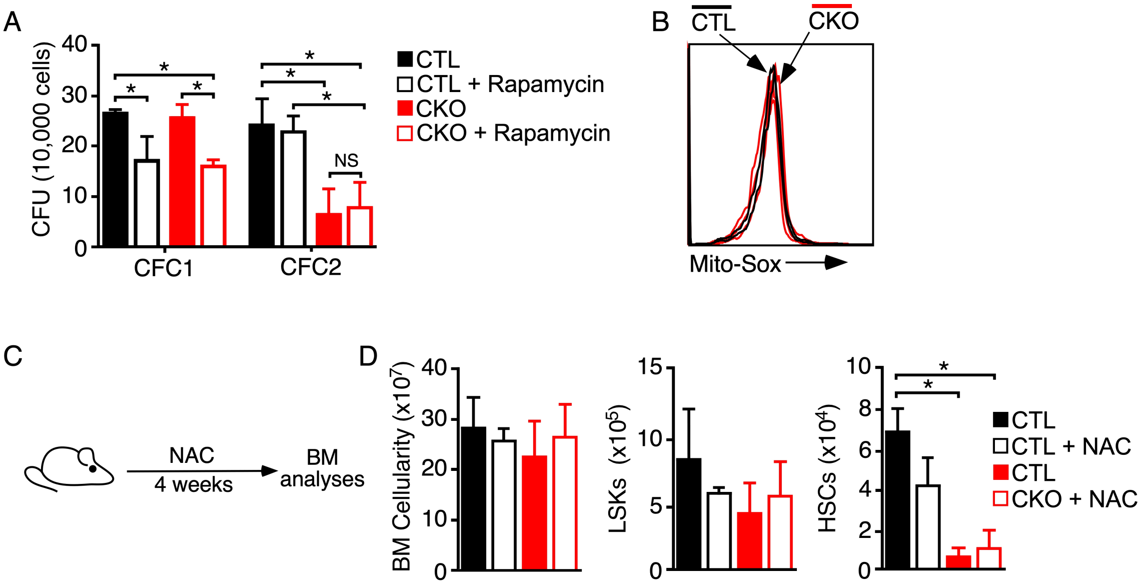

Figure S3. Rapamycin or N-acetyl-L-cysteine (NAC) do not rescue defects resulting from *Cited2* deficiency. (A) BM cells from 8- to 10-week-old *Cited2*^{CKO} and *Cited2*^{CTL} mice (n=4 per genotype) were placed in colony forming cell (CFC) assays in the presence and absence of 1μM rapamycin. CFC1 colonies were counted 10 days after plating, and re-plated into CFC2. CFC2 colonies were counted after 10 days in culture. Data are mean ± SEM. *, $P < 0.05$ (**B**) ROS levels in HSCs from *Cited2*^{CKO} and *Cited2*^{CTL} mice measured by MitoSOX (mitochondrial superoxide–sensitive fluorophore). (**C**) Experimental design. 9-week-old mice received 30 mg/mL of NAC in drinking water for 4 weeks. After 4 weeks of NAC administration, BM was analysed by FACS. (**D**) The graphs show total BM cellularity and total numbers in BM LSK cells and HSCs of *Cited2*^{CKO} and *Cited2*^{CTL} mice (n=4-6). Data are mean ± SEM. *, $P < 0.05$

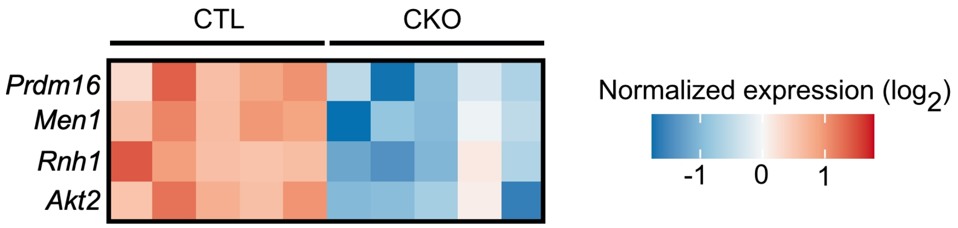

Figure S4. Decreased expression of *Prdm16*, *Men1*, *Rnh1* and *Akt2* in HSCs lacking *Cited2*. Heatmaps of significantly dysregulated genes (FDR < 0.05) essential for HSC functions in *Cited2*^{CKO} compared with *Cited2*^{CTL} HSCs (n=5).

Supplemental Experimental Procedures

Flow cytometry

Briefly, BM cells were obtained by crushing tibias and femurs with a pestle and mortar. Spleen cells were obtained by mashing the tissue through a 70μm strainer. Single cell suspensions from BM, spleen or PB were incubated in Fc block and then stained with antibodies. For HSC analyses, following incubation with Fc block, unfractionated BM cell suspensions were stained with lineage markers containing biotin-conjugated anti-CD4, anti-CD5, anti-CD8a, anti-CD11b, anti-B220, anti-Gr-1 and anti-Ter119 antibodies together with APC or APC/Cy7-conjugated anti-c-Kit, APC/Cy7- or PB-conjugated anti-Sca-1, PE-conjugated anti-CD48 and PE-Cy7-conjugated anti-CD150, and for some analysis APC-conjugated anti-CD135 and FITC-conjugated anti-CD34 antibodies were also added. For analysis of committed progenitor cells unfractionated BM cell suspensions were stained with lineage markers containing biotin-conjugated anti-CD4, anti-CD5, anti-CD8a, anti-CD11b, anti-B220 and anti-Gr-1 antibodies together with BV711-conjugated anti-c-Kit, APCCy7-conjugated CD16/32, APC-conjugated CD41, PE-conjugated CD105, BV421-conjugated CD127, PE-conjugated CD135 (Flt3), FITC-conjugated Ter119. Biotin-conjugated antibodies were then stained with Pacific Blue-conjugated or PerCP-conjugated streptavidin. To distinguish CD45.2^{+}-donor derived HSCs in recipient mice, FITC-conjugated anti-CD45.1 and Pacific Blue-conjugated anti-CD45.2 antibodies were included in the antibody cocktail described above. The multilineage reconstitution of recipient mice was determined by staining the BM or PB cell suspensions of the recipient mice with FITC-conjugated anti-CD45.1, Pacific Blue-conjugated anti-CD45.2, PE-conjugated anti-CD4 and -CD8a, PE/Cy7-conjugated anti-Gr-1, APC-conjugated anti-CD11b, APC-Cy7-conjugated anti-CD19 and anti-B220). For cell cycle analyses, cells were further stained with FITC-conjugated Ki67 and DAPI. For analyses of apoptosis, cells were further stained with FITC-conjugated Annexin-V and DAPI. In all analyses, 7-AAD or DAPI were used for dead cell exclusion. Flow cytometry analyses were performed using a LSRFortessa (BD). Cell sorting was performed on a FACSAria Fusion (BD). Analysis was done using FlowJo.
