# Supplementary material for: CITED2 coordinates key hematopoietic regulatory pathways to maintain the HSC pool in both steady-state hematopoiesis and transplantation
Source: Stem Cell Reports. 2021 Oct 28;16(11):2784–97. doi: 10.1016/j.stemcr.2021.10.001 (PMC8581166; doi:10.1016/j.stemcr.2021.10.001)
Supplement: Document S2. Article plus supplemental information [file mmc2.pdf]

Stem Cell Reports
Article
OPEN ACCESS

CITED2 coordinates key hematopoietic regulatory pathways to maintain the HSC pool in both steady-state hematopoiesis and transplantation

Hannah Lawson,[1,2] Louie N. van de Lagemaat,[1,2] Melania Barile,[3] Andrea Tavosanis,[1] Jozef Durko,[1] Arnaud Villacreces,[2] Aarushi Bellani,[1] Christopher Mapperley,[1] Elise Georges,[1] Catarina Martins-Costa,[2] Catarina Sepulveda,[2] Lewis Allen,[1] Joana Campos,[1] Kirsteen J. Campbell,[4] Dónal O'Carroll,[2] Berthold Göttgens,[3] Suzanne Cory,[5] Neil P. Rodrigues,[6] Amelie V. Guitart,[2,7,8,*] and Kamil R. Kranc[1,8,*]

[1]Laboratory of Haematopoietic Stem Cell & Leukaemia Biology, Centre for Haemato-Oncology, Barts Cancer Institute, Queen Mary University of London, London EC1M 6BQ, UK
[2]Centre for Regenerative Medicine, University of Edinburgh, Edinburgh EH16 4UU, UK
[3]Department of Haematology, Wellcome and Medical Research Council Cambridge Stem Cell Institute, Jeffrey Cheah Biomedical Centre, Cambridge Biomedical Campus, University of Cambridge, Cambridge CB2 0AW, UK
[4]CRUK Beatson Institute, Glasgow, UK
[5]The Walter and Eliza Hall Institute of Medical Research, Melbourne, Australia
[6]European Cancer Stem Cell Research Institute, Cardiff University, School of Biosciences, Cardiff CF24 4HQ, UK
[7]Université de Bordeaux, Institut National de la Santé et de la Recherche Médicale INSERM U1035, 33000 Bordeaux, France
[8]These authors contributed equally
*Correspondence: amelie.guitart@u-bordeaux.fr (A.V.G.), kamil.kranc@qmul.ac.uk (K.R.K.)
https://doi.org/10.1016/j.stemcr.2021.10.001

SUMMARY

Hematopoietic stem cells (HSCs) reside at the apex of the hematopoietic differentiation hierarchy and sustain multilineage hematopoiesis. Here, we show that the transcriptional regulator CITED2 is essential for life-long HSC maintenance. While hematopoietic-specific *Cited2* deletion has a minor impact on steady-state hematopoiesis, *Cited2*-deficient HSCs are severely depleted in young mice and fail to expand upon aging. Moreover, although they home normally to the bone marrow, they fail to reconstitute hematopoiesis upon transplantation. Mechanistically, CITED2 is required for expression of key HSC regulators, including GATA2, MCL-1, and PTEN. Hematopoietic-specific expression of anti-apoptotic MCL-1 partially rescues the *Cited2*-deficient HSC pool and restores their reconstitution potential. To interrogate the Cited2→Pten pathway in HSCs, we generated *Cited2*;*Pten* compound heterozygous mice, which had a decreased number of HSCs that failed to reconstitute the HSC compartment. In addition, CITED2 represses multiple pathways whose elevated activity causes HSC exhaustion. Thus, CITED2 promotes pathways necessary for HSC maintenance and suppresses those detrimental to HSC integrity.

INTRODUCTION

Hematopoiesis is a dynamic and essential process, with the capacity to meet a large demand for differentiated blood cells (~10^{11} cells per day in humans) (Sender and Milo, 2021). Hematopoiesis critically depends on a pool of bone marrow (BM)-resident adult hematopoietic stem cells (HSCs) at the apex of the hematopoietic differentiation hierarchy, which possess unique self-renewal capacity and multilineage differentiation potential (McCracken et al., 2016). The strict regulation of survival, quiescence, self-renewal, and differentiation in HSCs is essential for life-long maintenance of their pool. While much progress has been made in identifying individual pathways that suppress or promote these fates, the key regulators which coordinate these pathways to maintain the HSC pool both in the steady state and under conditions of physiologic stress remain poorly understood.

CITED2 (CBP/p300-interacting-transactivator-with-an ED-rich-tail 2) is a transcriptional regulator that co-activates or represses multiple transcription factors, including AP-2 (Bamforth et al., 2001), HIF-1alpha (Bhattacharya et al., 1999), PPAR-α (Tien et al., 2004), SMAD2/3 (Chou and Yang, 2006), and c-MYC (Chou et al., 2012) to regulate fundamental cellular processes, such as proliferation, metabolism, differentiation, migration, and autophagy. Consistent with its ubiquitous expression and pleiotropic impact on diverse transcription factors, CITED2 is essential for embryonic development, including fetal liver hematopoiesis (Bamforth et al., 2001, 2004; Chen et al., 2007; Weninger et al., 2005; Withington et al., 2006; Yin et al., 2002), ESC biology (Kranc et al., 2015; Li et al., 2012), adult tissue functions (Kim et al., 2018; Lee et al., 2009; Liu et al., 2019), cellular proliferation (Kranc et al., 2003), and cancer progression (Fernandes et al., 2020). Thus, CITED2 is an important regulator of diverse molecular, cellular, and developmental processes.

A growing body of evidence indicates that CITED2 is a key regulator of adult HSC biology (Du et al., 2012, 2014; Korthuis et al., 2015; Kranc et al., 2009). We previously reported that inducible *Mx1-Cre*-mediated deletion of *Cited2* (in which *Mx1-Cre* is induced by poly(I:C)-stimulated IFN-α production) results in a rapid loss of HSCs via apoptosis and a resultant BM failure (Kranc et al., 2009). In

This is an open access article under the CC BY license (http://creativecommons.org/licenses/by/4.0/).

this context, the significant loss of the HSC pool upon inducible *Cited2* deletion is at least in part caused by upregulation of the p19^{ARF}-p53 pathway, as genetic ablation of *Cdkn2a* (encoding p16^{INK4A} and p19^{ARF}) or *Trp53* (encoding p53) rescues depletion of *Cited2*-deficient HSCs. Another study (Du et al., 2012) employing a different *Cited2* floxed allele, also demonstrated that poly(I:C)-inducible *Mx1-Cre*-mediated *Cited2* deletion results in loss of HSCs (by affecting their quiescence and survival), compromises their reconstitution potential, and leads to a rapid BM failure upon myelotoxic stress. In this study, loss of quiescence, but not increased apoptosis, upon inducible *Cited2* deletion is mediated at least in part by HIF-1alpha, as *Hif1a* deletion partially restores impaired quiescence of HSCs lacking *Cited2* and improves their ability to reconstitute the HSC compartment upon transplantation (Du et al., 2012). Additional analyses of *Cited2*-deficient HSCs indicated alterations in HSC metabolism, namely a decrease in glycolytic flux and an increase in mitochondrial activity (Du et al., 2014), a state associated with a decline in HSC function (Lawson et al., 2021; Wang et al., 2014). However, given that the previous studies outlined above involved poly(I:C) administration, which is known to induce interferon response, proinflammatory pathways, and subsequent over-proliferation in HSCs, the functional significance of CITED2 in maintenance of the HSC pool under steady-state conditions is yet to be investigated.

Here, we reveal that, under steady-state conditions, CITED2 is largely dispensable for unperturbed long-term multilineage hematopoiesis, but is critically required for the maintenance of the HSC pool and HSC function post-transplantation. We found that CITED2 represses pathways that inhibit HSC maintenance and promotes pathways required for HSC integrity. Notably, our functional genetic approaches found that key regulators of HSC maintenance, namely MCL-1 and PTEN, act downstream of CITED2 and mediate, at least in part, its critical role in regulating the HSC pool. Taken together, we propose that CITED2 coordinates multiple fundamental stem cell regulatory pathways to promote the maintenance of the HSC pool under steady-state conditions and upon transplantation.

RESULTS

Cited2 deletion does not derail normal steady-state hematopoiesis

To determine the expression of *Cited2* at different levels of the hematopoietic hierarchy, we sorted Lin^{-}Sca-1^{+}c-Kit^{+} (LSK) cells, LSKCD48^{-}CD150^{+} HSCs, Lin^{-}Sca-1^{-}c-Kit^{+} (LK) myeloid progenitors, and more-mature Lin^{-} and differentiated Lin^{+} hematopoietic cell populations, and performed qRT-PCR. *Cited2* was expressed in all compartments, with significantly higher expression in the HSC population compared with myeloid progenitors and more mature hematopoietic cell populations (Figure 1A). Furthermore, to compare the expression of *Cited2* in long-term HSCs (LSKCD34^{-}CD135^{-}), MPP1 (LSKCD34^{+}CD135^{-}CD150^{+}CD48^{-}), MPP2 (LSKCD34^{+}CD135^{-}CD150^{+}CD48^{+}), and MPP3 (LSKCD34^{+}CD135^{-}CD150^{-}CD48^{+}) populations, lymphoid-primed multipotent progenitors (LSKCD34^{+}CD135^{+}), which correspond to the MPP4 population, and CMP (LKCD34^{+}FcγRII/III^{low}), GMP (LKCD34^{+}FcγRII/III^{high}), and MEP (LKCD34^{-}FcγRII/III^{low}) compartments, we analyzed our SMART2-seq single-cell expression data in these populations (Nestorowa et al., 2016). *Cited2* was rather uniformly expressed across these populations (Figure 1B), with the highest expression in the HSC, GMP, and CMP compartments, and lowest in the MEP population. Moreover, we employed our 10× Genomics single-cell RNA sequencing (RNA-seq) data set (Dahlin et al., 2018) to compare *Cited2* expression between HSCs and committed progenitor cell compartments. *Cited2* was expressed highly in HSCs and committed megakaryocytic progenitors, but decreased in committed lymphoid, basophilic, neutrophilic, and erythroid progenitor cells (Figure 1C). Thus, *Cited2* is ubiquitously expressed in hematopoiesis, with robust high expression at the apex of the hematopoietic hierarchy, and lower expression in more differentiated cells.

Given that the functional significance of *Cited2* in unperturbed hematopoiesis remains poorly understood, we combined the *Cited2*^{fl} allele (Kranc et al., 2009; MacDonald et al., 2008) with *Vav-iCre* (de Boer et al., 2003) to generate *Cited2*$^{fl/fl}$;*Vav-iCre* (*Cited2*^{CKO}) mice (Figure 1D), where *Cited2* is deleted specifically from the hematopoietic system shortly after the emergence of HSCs. Consequently, *Cited2* expression was completely lost in BM c-Kit^{+} cells isolated from *Cited2*^{CKO} mice (Figure 1E). Surprisingly, *Cited2* deletion had no impact on animal viability, and all animals survived to adulthood without any obvious defects. Peripheral blood (PB) analyses of 8- to 12-week-old *Cited2*^{CKO} mice revealed unaffected WBC counts, with mild anemia and thrombocythemia (Figure 1F). Furthermore, *Cited2*^{CKO} mice displayed normal BM cellularity (Figure 1G) and unaffected numbers of differentiated myeloid and B lymphoid cells in the BM and spleens (Figure 1H), as well as normal distribution of T cells in the thymi and spleens (Figures S1 and 1I). *Cited2*^{CKO} mice also had normal myeloid and megakaryocytic progenitor cell numbers (Figure 1J). Notably, consistent with mild anemia (Figure 1F), mice lacking *Cited2* had significantly decreased numbers of BM erythroid

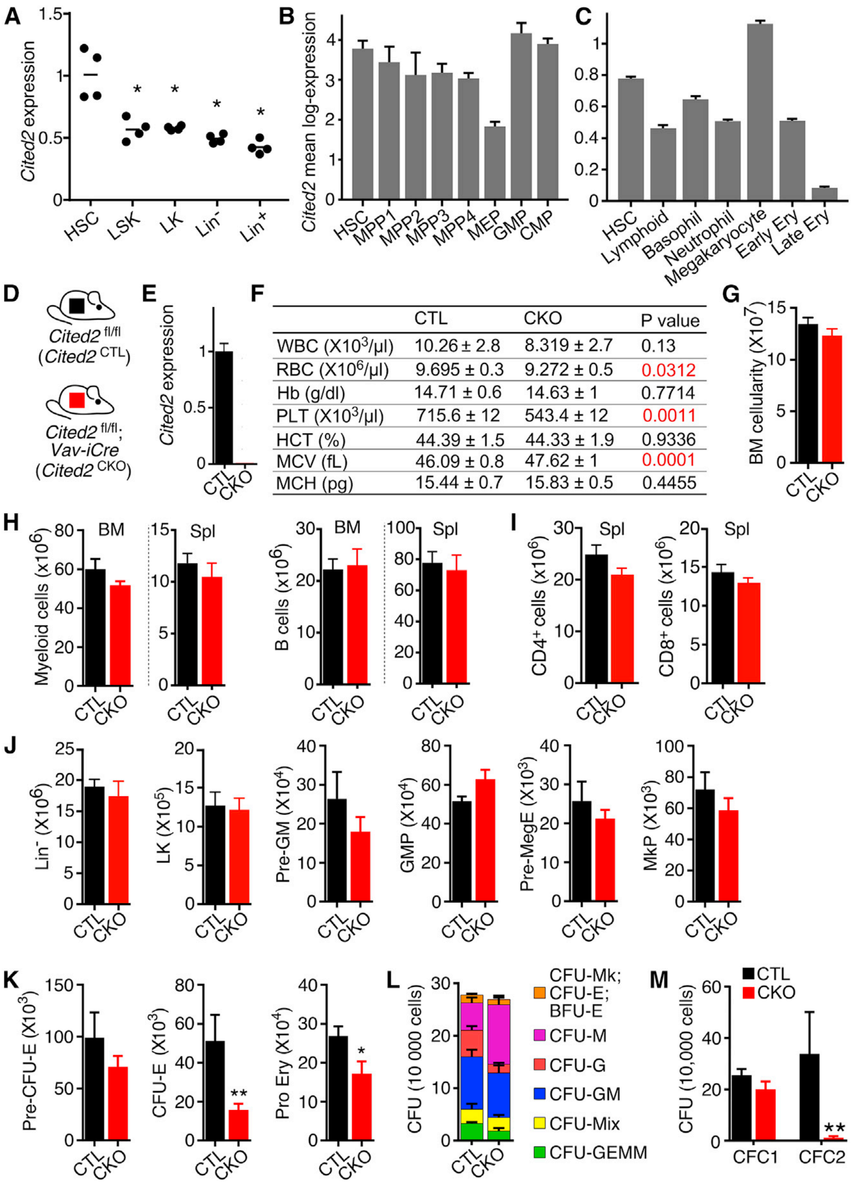

	CTL	CKO	P value
WBC (X10³/µl)	10.26 ± 2.8	8.319 ± 2.7	0.13
RBC (X10⁶/µl)	9.695 ± 0.3	9.272 ± 0.5	0.0312
Hb (g/dl)	14.71 ± 0.6	14.63 ± 1	0.7714
PLT (X10³/µl)	715.6 ± 12	543.4 ± 12	0.0011
HCT (%)	44.39 ± 1.5	44.33 ± 1.9	0.9336
MCV (fL)	46.09 ± 0.8	47.62 ± 1	0.0001
MCH (pg)	15.44 ± 0.7	15.83 ± 0.5	0.4455

Figure 1. Hematopoiesis-specific *Cited2* deletion does not derail multilineage hematopoiesis

(A) *Cited2* mRNA levels in cells isolated from 8-week-old C57BL/6 mice (n = 4).

(B) *Cited2* expression in different hematopoietic compartments determined using single-cell SMART2-seq.

(C) *Cited2* expression in HSCs and committed progenitor cell compartments determined by 10× Genomics single-cell RNA-seq.

(D) *Cited2*$^{fl/fl}$ mice were bred to *Vav-iCre* mice to generate *Cited2*$^{fl/fl}$;*Vav-iCre* (*Cited2*^{CKO}) mice. *Cited2*$^{fl/fl}$ mice were used as controls (*Cited2*^{CTL}).

(E) *Cited2* expression in c-Kit$^+$ cells from BM of *Cited2*^{CTL} and *Cited2*^{CKO} mice (n = 4).

(F) PB counts of 8- to 10-week-old mice (n = 14).

(G) Total BM cellularity (n = 6–9).

(H) Total number of differentiated myeloid (Gr-1$^+$Mac-1$^+$) and B cells (CD19$^+$B220$^+$) in BM and spleen (Spl) (n = 6–9).

(I) Total number of mature CD4$^+$ and CD8$^+$ T cells in spleens (n = 6–9).

(J) Total number of Lin$^-$ cells, LK cells, pre-GM (LKCD41$^-$FcγRII/III$^-$CD150$^-$CD105$^-$), GMP (LKCD41$^-$FcγRII/III$^+$), Pre-MegE (LKCD41$^-$FcγRII/III$^-$CD150$^-$CD105$^+$), MkP (LKCD41$^+$CD150$^+$) cells (n = 5).

(K) Total number of Pre-CFU-E (LKCD41$^-$FcγRII/III$^-$CD150$^+$CD105$^+$), CFU-E (LKCD41$^-$FcγRII/III$^-$CD150$^+$CD105$^+$CD71$^+$Ter119$^-$), and Pro Ery (LKCD41$^-$FcγRII/III$^-$CD150$^+$CD105$^+$CD71$^+$Ter119$^+$) cells (n = 5).

(L) Colony-forming unit (CFU) assay performed with 10^4 BM cells from 8- to 10-week-old mice. CFU-megakaryocyte (CFU-Mk), CFU-erythroid (CFU-E), burst-forming unit (BFU-E), CFU-granulocyte (CFU-G), CFU-monocyte/macrophage (CFU-M), CFU-granulocyte, and monocyte/macrophage (CFU-GM); at least three lineages (CFU-Mix), CFU with all four lineages, granulocyte, erythroid, monocyte/macrophage, and megakaryocyte (CFU-GEMM) (n = 7).

(M) Colony counts of primary and secondary cultures (n = 7). For (B), (C), and (F)–(M), data are mean ± SEM. * $p < 0.05$, ** $p < 0.01$

progenitors (Figure 1K). Finally, colony-forming cell (CFC) assays showed normal differentiation potential of *Cited2*^{CKO} BM cells (Figures 1L and 1M). Importantly, however, *Cited2*-deficient cells failed to form secondary colonies after replating, suggesting that *Cited2* is required for propagation or self-renewal of progenitor cells (Figure 1M). Taken together, while *Cited2* deletion compromises erythroid progenitors and causes mild anemia, it is otherwise not essential for normal steady-state multilineage hematopoiesis in young adult mice.

Cited2 is required for the maintenance of the HSC pool under steady-state conditions and its expansion upon aging

We next determined the impact of *Cited2* deletion on HSCs and primitive progenitor cells. We found that the total number of LSK cells (the compartment comprising HSCs and functionally distinct lineage-biased multipotent progenitors [MPP1-4]) was not affected in young 8- to 12-week-old *Cited2*^{CKO} mice (Figure 2A). Markedly, however, mice lacking *Cited2* displayed significant depletion of

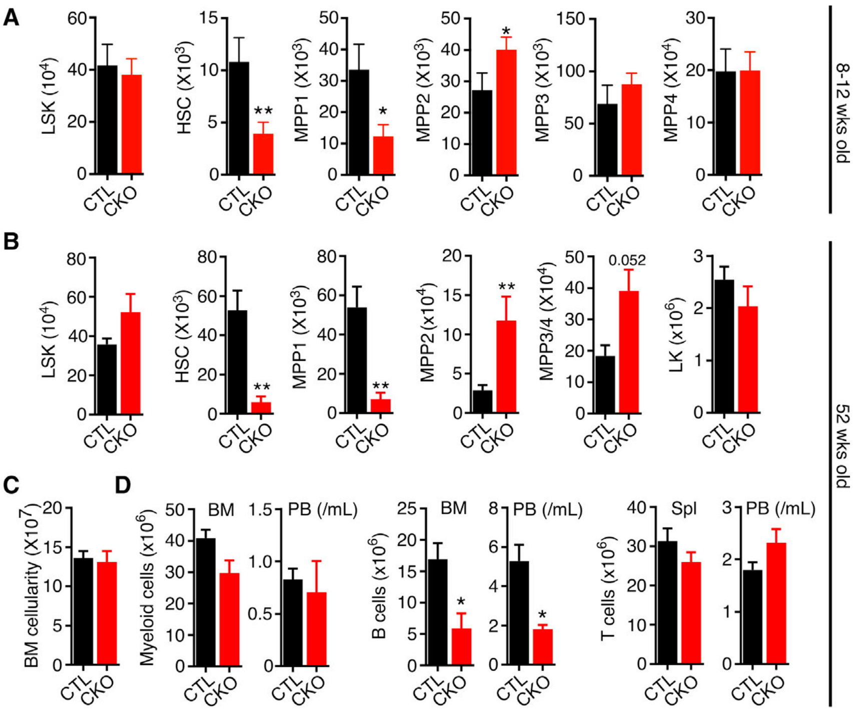

E

	CTL	CKO	P value
WBC (X10³/μl)	9.893±3.881	5.952± 2.348	0.1522
RBC (X10⁶/μl)	9.829± 0.96	7.997± 1.463	0.0307
Hb (g/dl)	14.21±1.131	11.94±2.188	0.0542
PLT (X10³/μl)	1049±257	693± 281	0.0640
HCT (%)	41.6±6.504	34.40±9.762	0.4135

Figure 2. *Cited2* is required for the maintenance of the HSC pool and its expansion upon aging

(A) Total number of LSK cells, LSKCD48$^-$CD150$^+$Flt3$^-$CD34$^-$ HSCs, LSKCD48$^-$CD150$^+$Flt3$^-$CD34$^+$ MPP1, LSKCD48$^+$CD150$^+$Flt3$^-$ MPP2, LSKCD48$^+$CD150$^-$Flt3$^-$ MPP3, and LSKCD48$^+$CD150$^-$Flt3$^+$ MPP4 populations in BM of 8- to 10-week-old mice (n = 6–9).

(B) Total number of LSK cells, HSCs, MPP1, MPP2, MPP3/MPP4, and LK cells in BM in 52-week-old mice (n = 6).

(C) BM cellularity in 52-week-old mice (n = 6–9).

(D) Total number of differentiated myeloid cells (Gr-1$^+$Mac-1$^+$) and B cells (CD19$^+$B220$^+$) in BM, spleen (Spl) and PB in 52-week-old mice (n = 6–9).

(E) PB counts in 52-week-old *Cited2*^{CTL} and *Cited2*^{CKO} mice (n = 6–9). For (A)–(E), data are mean ± SEM. *$p < 0.05$, **$p < 0.01$.

HSCs and the most primitive progenitors (i.e., MPP1 population), with an increase in MPP2 population and unchanged numbers of the MPP3-4 populations. Thus, despite a select reduction in absolute HSC and MPP1 cell numbers, *Cited2*^{CKO} mice sustain largely unaffected unperturbed steady-state multilineage hematopoiesis.

Next, we investigated the impact of prolonged *Cited2* deficiency on long-term HSC maintenance during unperturbed hematopoiesis. We aged mouse cohorts for 52 weeks and found that *Cited2* deficiency had no impact on mouse survival (data not shown). Consistent with physiological HSC aging, during which the HSC pool undergoes expansion (Geiger et al., 2013), we found that aging *Cited2*^{CTL} mice had approximately 5-fold more HSCs compared with 8- to 12-week-old *Cited2*^{CTL} mice (Figures 2A and 2B). Strikingly however, *Cited2*^{CKO} HSCs failed to expand upon aging and remained severely depleted compared with *Cited2*^{CTL} HSCs (Figure 2B). Furthermore, while the MPP1 population also remained depleted, the numbers of MPP2 and MPP3/4 populations were increased in 52-week-old *Cited2*^{CKO} mice. Aging *Cited2*^{CKO} mice had normal BM cellularity and unaffected numbers of primitive and differentiated myeloid cells and T cells, while B cell numbers were decreased (Figures 2C and 2D). Apart from anemia and a significant drop in B cells, PB analyses showed no other major abnormalities (Figures 2D and 2E). Taken together, *Cited2* is essential for both the maintenance of HSCs and their expansion during physiological aging, but remarkably, despite this, *Cited2* is not critical for long-term multilineage hematopoiesis. Notably, these data also reveal the requirement for CITED2 in the maintenance of the B cell lineage, meriting further investigations.

Cited2 is essential for post-transplantation HSC functions

Given that *Cited2*^{CKO} mice do not display any severe hematopoietic defects despite a substantial depletion of the HSC pool, we next investigated the multilineage reconstitution capacity of *Cited2*^{CKO} HSCs. We competitively transplanted 100 HSCs from 8- to 12-week-old *Cited2*^{CKO} and *Cited2*^{CTL} mice into lethally irradiated recipients (Figure 3A). *Cited2*-deficient HSCs failed to reconstitute hematopoiesis (Figure 3B), and were unable to contribute to the LSK pool of recipient mice (Figure 3C). Given that HSCs

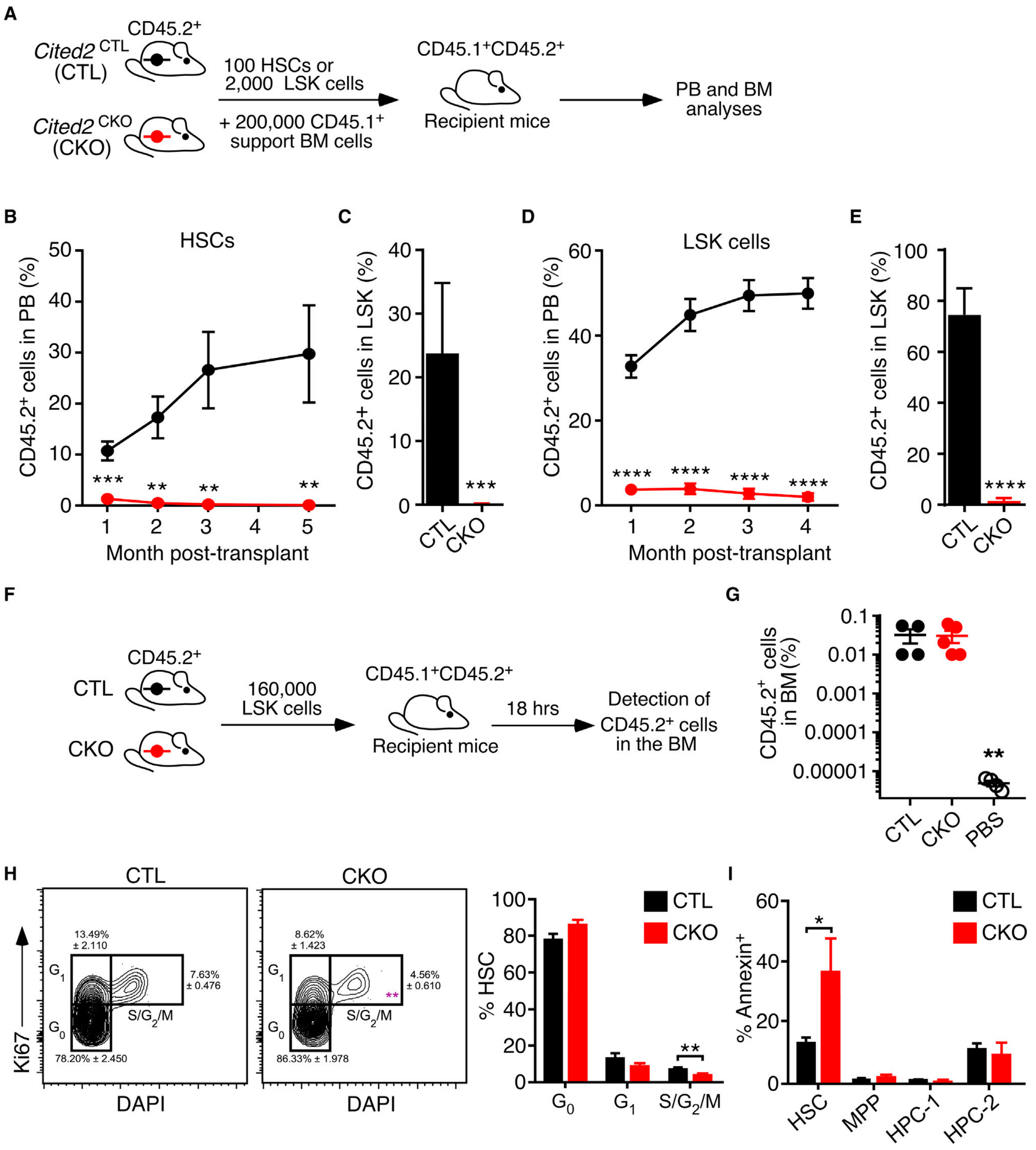

Figure 3. HSCs critically require *Cited2* to reconstitute hematopoiesis upon transplantation

(A) Transplantation assay: 100 HSCs or 2,000 LSK cells sorted from *Cited2*^{CTL} and *Cited2*^{CKO} mice were transplanted into lethally irradiated recipients together with 200,000 support CD45.1^{+} total BM cells.

(B and C) Percentage of donor-derived CD45.2^{+} cells in (B) PB and (C) the LSK cell compartment of recipient mice following transplantation of 100 HSCs (n = 5 recipients per donor; 2 donors per genotype).

(D and E) Percentage of donor-derived CD45.2^{+} cells in (D) PB and (E) the LSK compartment of the recipient mice following transplantation of 2,000 LSK cells (n = 4 recipients per donor; 4 donors per genotype).

(legend continued on next page)

lacking *Cited2* have no reconstitution activity but that *Cited2*^{CKO} mice are able to sustain hematopoiesis, we next asked whether the reconstitution activity is contained within the LSK compartment. We competitively transplanted LSK cells from 8- to 12-week-old *Cited2*^{CKO} and *Cited2*^{CTL} mice (together with 200,000 unfractionated CD45.1^{+} BM cells) and found that they also dramatically failed to reconstitute hematopoiesis (Figure 3D), and did not contribute to the LSK pool of the recipients (Figure 3E). Given that both *Cited2*-deficient HSCs and LSK cells fail to reconstitute hematopoiesis, we asked whether *Cited2* loss impacts on the ability of HSCs to home to the BM. We transplanted LSK cells from *Cited2*^{CKO} and *Cited2*^{CTL} mice into irradiated recipients (Figure 3F), and found equal numbers of control and *Cited2*-deficient CD45.2^{+} cells 18 h after injection, indicating that LSK cells lacking *Cited2* are able to home to the BM as efficiently as their *Cited2*^{CTL} counterparts (Figure 3G). Therefore, although multilineage steady-state hematopoiesis is maintained in *Cited2*^{CKO} mice, neither HSC nor LSK populations lacking *Cited2* have the ability to repopulate hematopoiesis upon transplantation. Thus, although *Cited2*-deficient HSCs home to the BM, they critically require *Cited2* to contribute to and sustain hematopoiesis upon transplantation.

HSCs lacking *Cited2* remain quiescent but display increased apoptotic rate

Depletion of HSCs and their failure to sustain hematopoiesis upon serial transplantation frequently result from a loss of HSC quiescence or increased apoptosis (Rossi et al., 2012). Thus, we investigated whether the reduction of HSCs and their reconstitution failure upon *Cited2* deletion is associated with changes in these HSC fates. To determine the cell-cycle status of *Cited2*-deficient HSCs, we employed Ki67 and DAPI staining, which showed no differences in quiescence between *Cited2*^{CTL} and *Cited2*^{CKO} HSCs (Figure 3H). Notably, however, the percentage of actively cycling HSCs (i.e., those in S/G2/M phases) was decreased in the absence of *Cited2* (Figure 3H). Furthermore, to determine the rate of cell death in *Cited2*-deficient HSCs, we used Annexin V staining. *Cited2*-deficient HSCs, but not primitive progenitors, displayed a significantly increased rate of apoptosis compared with their *Cited2*^{CTL} counterparts (Figure 3I). Therefore, depletion of *Cited2*-deficient HSCs, and their inability to expand over time and reconstitute hematopoiesis upon transplantation likely result, at least in part, from a decrease in HSC cycling and an increase in their apoptosis.

Cited2 maintains HSCs by regulating *Mcl1* expression

To understand the mechanisms through which *Cited2* loss depletes the HSC pool in *Cited2*^{CKO} mice and compromises HSC functions upon transplantation, we performed gene expression analyses in HSCs sorted from *Cited2*^{CKO} and *Cited2*^{CTL} mice. Interestingly, we found that the expression of key HSC regulators, including *Mcl1*, *Pten*, and *Gata2* (Menendez-Gonzalez et al., 2019; Opferman et al., 2005; Yilmaz et al., 2006), was decreased in *Cited2*-deficient HSCs (Figure 4A), suggesting that CITED2 may control several pathways important for HSC maintenance.

Myeloid cell leukemia 1 (MCL-1) is a pro-survival BCL-2 protein family member, whose *Mx1-Cre*-mediated deletion results in severe loss of HSCs and BM failure (Opferman et al., 2005), thus resembling the phenotype resulting from *Mx1-Cre*-mediated *Cited2* deletion (Kranc et al., 2009). Moreover, MCL-1 is also required for self-renewal of human HSCs (Campbell et al., 2010a). Given that *Mcl1* expression was decreased in *Cited2*-deficient HSCs, we sought to determine whether MCL-1 can rescue HSC defects resulting from *Cited2* deletion *in vivo*. We bred *Cited2*^{CKO} mice to *VavP-Mcl1* transgenic mice (Campbell et al., 2010b), which overexpresses *Mcl1* specifically within hematopoietic system, under the control of *Vav* regulatory elements (Figure 4B). Interestingly, *Mcl1* overexpression in *Cited2*^{CKO} mice (i.e., *Cited2*^{CKO};*Mcl1* mice) resulted in a partial rescue of the HSC and MPP1 cell pools compared with *Cited2*^{CKO} mice (Figure 4C). To investigate if *Mcl1* overexpression can support *Cited2*-deficient HSCs to reconstitute hematopoiesis, we competitively transplanted 100 HSCs from *Cited2*^{CTL}, *Cited2*^{CTL};*Mcl1*, *Cited2*^{CKO}, and *Cited2*^{CKO};*Mcl1* mice into lethally irradiated recipient mice (Figure 4D). Significantly, while *Cited2*-deficient HSCs failed to reconstitute recipient mice, HSCs from *Cited2*^{CKO};*Mcl1* mice successfully repopulated recipients, comparable with the reconstitution potential of HSCs from *Cited2*^{CTL} and *Cited2*^{CTL};*Mcl1* mice (Figures 4E and 4F). No statistically significant differences were found in repopulation potential between HSCs from *Cited2*^{CTL} and *Cited2*^{CTL};*Mcl1* mice (Figures 4E and 4F). Consistent with these data, HSCs from *Cited2*^{CKO};*Mcl1* mice contributed significantly more to the total BM and HSC compartments of recipient mice compared with *Cited2*^{CKO} HSCs, which failed to

(F) Homing assay: 160,000 LSK cells were injected into irradiated CD45.1^{+}-recipient mice and analyzed 18 h later.
(G) Percentage of donor-derived CD45.2^{+} cells in BM (n = 4–5).
(H) Percentage of HSCs from 8- to 10-week-old *Cited2*^{CKO} and *Cited2*^{CTL} mice in the G_0 (DAPI^{-}Ki67^{-}), G_1 (DAPI^{-}Ki67^{+}), and S/G_2/M (DAPI^{+}Ki67^{+}) phases of the cell cycle (n = 3).
(I) Percentage of Annexin V^{+} cells in the HSC, MPP, HPC-1, and HPC-2 cell compartments in BM of *Cited2*^{CKO} and *Cited2*^{CTL} mice (n = 3). For (B)–(E) and (G)–(I), data are mean ± SEM. *$p < 0.05$, **$p < 0.01$, ***$p < 0.001$, ****$p < 0.0001$.

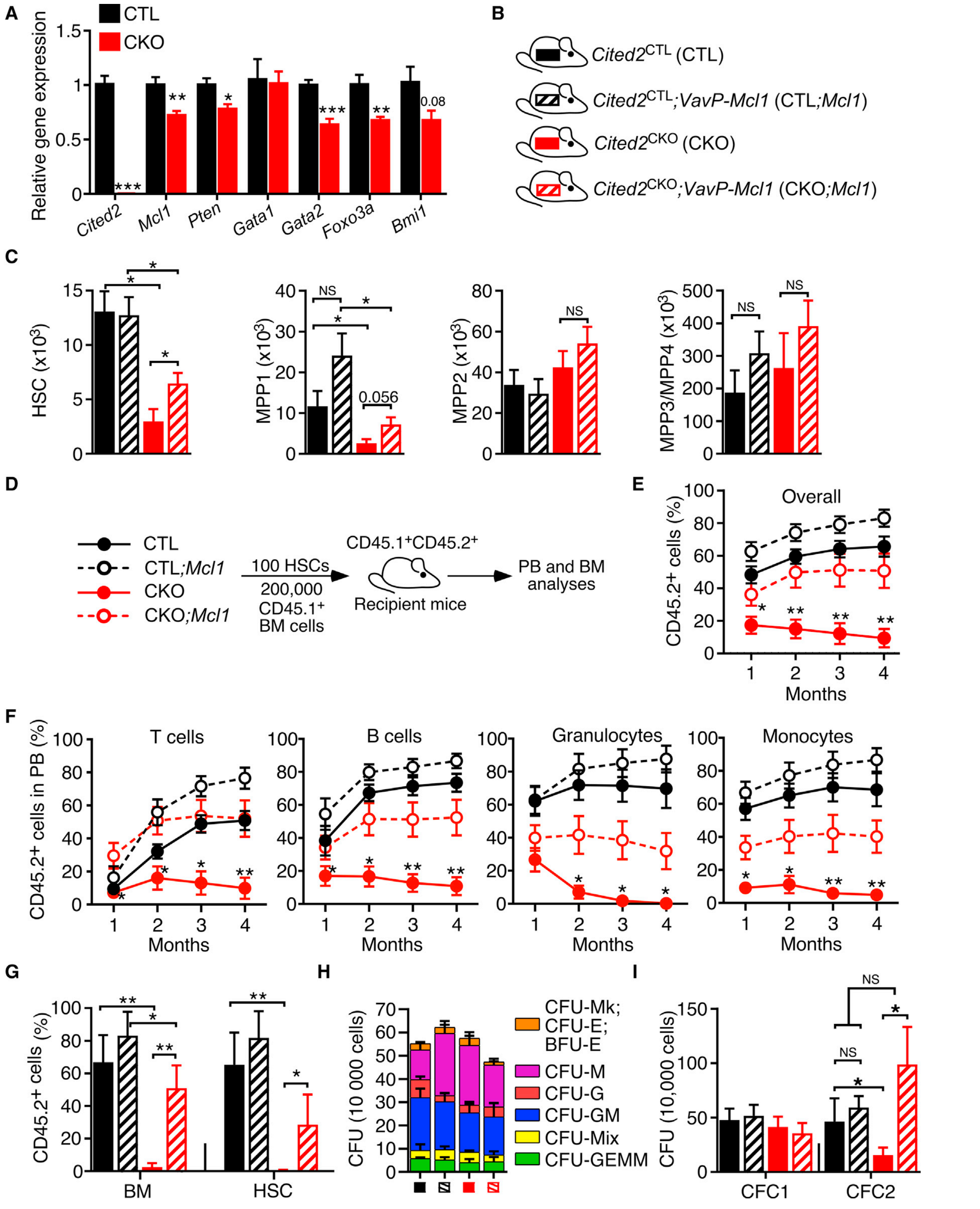

Figure 4. *Mcl1* partially restores normal HSC maintenance and function of *Cited2*-deficient HSCs

(A) Expression of *Cited2*, *Mcl1*, *Pten*, *Gata1*, *Gata2*, *Foxo3a*, and *Bmi1* in LSK cells sorted from 8-week-old *Cited2*^{CTL} and *Cited2*^{CKO} mice (n = 4).

(B) Schematic representation of experimental mouse cohorts; *Cited2*^{CTL}, *Cited2*^{CTL};*Mcl1*, *Cited2*^{CKO}, and *Cited2*^{CKO};*Mcl1*.

(legend continued on next page)

efficiently reconstitute recipients (Figure 4G). Finally, BM cells from *Cited2*^{CTL}, *Cited2*^{CTL};*Mcl1*, *Cited2*^{CKO}, and *Cited2*^{CKO};*Mcl1* mice efficiently generated primary colonies (Figure 4H), and while *Cited2*-deficient cells failed to replate secondary colonies, strikingly, *Cited2*^{CKO};*Mcl1* cells efficiently produced comparable colony numbers to control cells (Figure 4I). Therefore, *Mcl1* acts downstream of *Cited2* *in vivo* and at least in part mediates CITED2 functions in sustaining the HSC pool under steady-state hematopoiesis and promoting their long-term reconstitution capacity.

Cited2 regulates *Pten* to maintain the HSC pool

PTEN is required for cell-autonomous HSC maintenance (Yilmaz et al., 2006; Zhang et al., 2006) and its deletion results in loss of adult HSC function through activation of mTORC2-dependent signaling (Magee et al., 2012). Given that *Pten* expression was decreased in *Cited2*-deficient HSCs (Figure 4A), we sought to genetically interrogate the putative CITED2→PTEN axis. We took advantage of the principle that if genes act in common pathways they should genetically interact, i.e., the compound heterozygosity should generate phenotypes not observed in mice heterozygous for a single gene of interest (Vidal et al., 2011). We investigated whether combined *Pten* and *Cited2* heterozygosity causes loss of HSC functions compared with *Pten* or *Cited2* heterozygosity alone. We generated *Cited2*$^{+/fl}$;*Pten*$^{+/fl}$;*Vav-iCre* (*Cited2*^{Het};*Pten*^{Het}), *Cited2*^{Het}, *Pten*^{Het}, and control mice (Figure S2A). We found that *Cited2*^{Het} and *Pten*^{Het} mice had normal BM cellularity and largely unaffected numbers of Lin^{-}, LK, and LSK cells within the BM (Figures S2B and S2C). However, while control, *Cited2*^{Het}, and *Pten*^{Het} mice had comparable numbers of HSCs, *Cited2*^{Het};*Pten*^{Het} mice displayed subtly but significantly decreased numbers of HSCs. To assess the repopulation capacity of *Cited2*^{Het};*Pten*^{Het} HSCs, we competitively transplanted HSCs of all relevant genotypes into lethally irradiated recipients (Figure S2D). While HSCs of all genotypes gave equal overall long-term reconstitution capacity in the PB compartment (Figure S2E), *Cited2*^{Het};*Pten*^{Het} HSCs had a slightly decreased capacity to contribute to the BM reconstitution of the recipient mice (Figure S2F). Moreover, *Cited2*^{Het};*Pten*^{Het} HSCs failed to contribute to the HSC compartments of the recipients (Figure S2G). Therefore, given that *Cited2*^{Het};*Pten*^{Het} HSCs sustain hematopoiesis upon transplantation, but fail to efficiently repopulate the HSC compartment of the recipients, the CITED2→PTEN axis may contribute to long-term HSC maintenance but is unlikely to be essential for the reconstitution potential of HSCs.

Cited2 deletion dysregulates multiple pathways whose strict control is required for HSC integrity

To further understand why *Cited2*-deficient HSCs undergo depletion and fail upon transplantation, we examined global gene expression in *Cited2*-deficient HSCs by RNA-seq. This analysis identified a number of dysregulated genes, with 119 upregulated and 143 downregulated genes (Figure 5A). Gene set enrichment analysis (GSEA) indicated a broad dysregulation of multiple pathways and processes, which was compatible with the loss of HSC function. Notably, we found that *Cited2*-deficient HSCs displayed activation of proinflammatory pathways, c-MYC and E2F targets, and K-RAS and mTORC1 signaling, whose elevated activity is known to lead to HSC exhaustion or loss of their reconstitution potential (Kim et al., 2017; Pietras, 2017; Sasine et al., 2018; Wilson et al., 2004; Yilmaz et al., 2006) (Figures 5B and 5C). Consistent with upregulation of E2F and c-MYC targets, analysis of proximal promoters of upregulated genes revealed the presence of E2F and c-MYC motifs, suggesting that CITED2 may repress E2Fs and c-MYC (Figure 5D). We also found that promoters of the upregulated genes were enriched for the motif of RUNX1 (Figure 5D), whose increased expression in adult HSCs results in loss of their reconstitution potential (Ichikawa et al., 2008).

Given that we observed activation of the mTORC1 signaling signature upon *Cited2* deletion in HSCs (Figures 5B and 5C), we asked whether rapamycin, a known mTORC1 inhibitor (Guertin and Sabatini, 2007), can rescue the replating defect resulting from *Cited2* deficiency. We serially plated BM cells from *Cited2*^{CTL} and *Cited2*^{CKO} mice into CFC assays, in the presence or absence of rapamycin. As expected, *Cited2*^{CTL} and *Cited2*^{CKO} cells generated primary colonies (Figure S3A). However, while *Cited2*^{CTL} cultures efficiently produced secondary colonies, *Cited2*^{CKO} cells displayed a replating defect, regardless of the presence or absence of rapamycin (Figure S3A). As such, we conclude that upregulation of the mTORC1

(C) Total number of HSC, MPP1, MPP2, and MPP3/MPP4 cell populations in BM of 8- to 10-week-old mice (n = 4–6 mice per genotype).
(D) Transplantation assay: 100 HSCs were transplanted into lethally irradiated recipients together with 200,000 unfractionated CD45.1^{+} BM cells.
(E) Percentage of donor-derived CD45.2^{+} cells in PB following transplantation (n = 4 recipients per donor; n = 2–3 donors).
(F) Percentage of donor-derived CD45.2^{+} cells overall in the monocyte, granulocyte, B cell, and T cell compartments of PB.
(G) Percentage of donor-derived CD45.2^{+} cells in total BM and HSC compartments of the recipient mice.
(H) CFU assay performed with 10^4 BM cells from 8- to 10-week-old mice (n = 5).
(I) Total CFC counts of primary and secondary cultures (n = 5). For (A), (C), and (E)–(I), data are mean ± SEM. $^{*}p < 0.05$, $^{**}p < 0.01$.

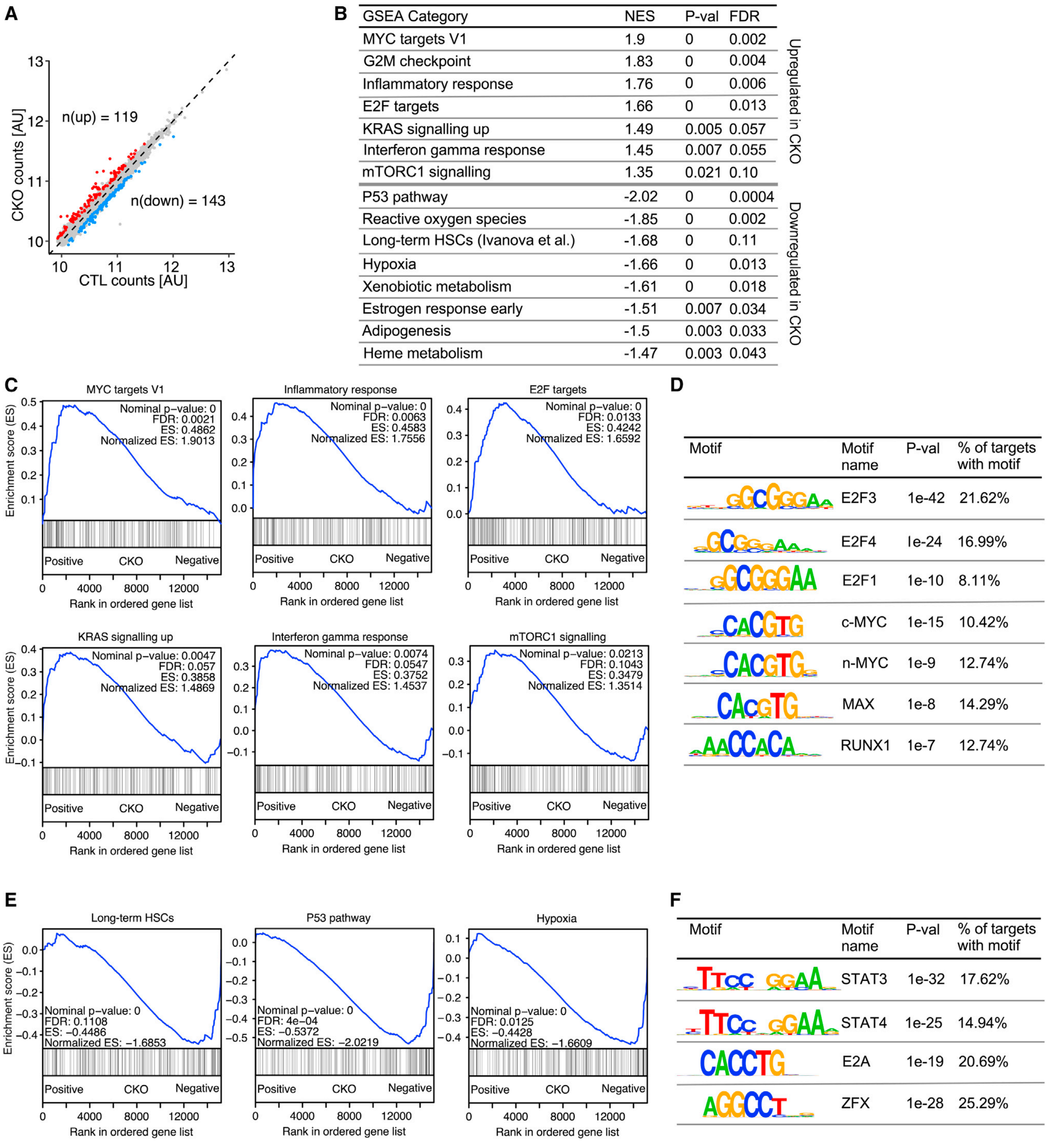

Figure 5. *Cited2*-deficient HSCs exhibit molecular signatures of functional HSC decline

(A) Expression scatterplot of *Cited2*^{CTL} versus *Cited2*^{CKO} HSCs from 8- to 12-week-old mice (n = 5). Transcripts significantly up- (red) and downregulated (blue) in *Cited2*^{CKO} are highlighted (FDR < 0.05 and FC > 20%).

(B) GSEA showing hallmark pathways up- and downregulated in *Cited2*^{CKO} HSCs.

(C) GSEA plots showing upregulated pathways in *Cited2*^{CKO} HSCs.

(D) DNA motifs of transcription factors enriched in proximal promoters (from −200 to +100 bp from transcription start site) of genes upregulated in *Cited2*^{CKO} HSCs.

(E) GSEA plots showing downregulated pathways in *Cited2*^{CKO} HSCs.

(F) DNA motif enrichments in proximal promoters of genes that are downregulated in *Cited2*^{CKO} HSCs.

pathway alone in *Cited2*-deficient cells is insufficient to elicit the observed phenotypes. The identification and dissection of upregulated pathways upon *Cited2* deletion, which act together to cause HSC depletion, merit future investigation.

We next focused on pathways that were downregulated in *Cited2*^{CKO} HSCs. Interestingly, *Cited2* loss led to an overall downregulation in genes whose expression is a hallmark of long-term HSC signature (Ivanova et al., 2002) (Figures 5B and 5E). *Cited2* loss also led to a decreased signature of the p53 pathway (Figures 5B and 5E), whose inhibition is detrimental to HSC integrity and function (Liu et al., 2009). *Cited2*^{CKO} HSCs displayed downregulation of the hypoxic signature (Figures 5B and 5E), implying their intrinsic inability to adapt to the physiologically hypoxic BM microenvironment where HSCs reside (Spencer et al., 2014). Moreover, we found that the reactive oxygen species (ROS) defense pathway was downregulated (Figure 5B). However, ROS levels were not elevated in *Cited2*^{CKO} HSCs (Figure S3B), and treatment of *Cited2*^{CKO} mice with the antioxidant N-acetyl-L-cysteine (NAC) did not rescue HSC depletion (Figures S3C and S3D), suggesting that ROS is unlikely to be responsible for the observed effects in HSCs lacking *Cited2*. Furthermore, in addition to the GSEA, our data revealed that several genes essential for HSC functions, including *Prdm16*, *Men1*, *Rnh1*, and *Akt2* were downregulated in *Cited2*^{CKO} HSCs (Figure S4) (Andina et al., 2019; Gudmundsson et al., 2020; Juntilla et al., 2010; Maillard et al., 2009). Finally, further interrogation of promoters of the downregulated genes revealed the presence of motifs for STAT3, STAT4, E2A, and ZFX transcription factors (Figure 5F), all of which are required for HSC maintenance and function (Galan-Caridad et al., 2007; Holmfeldt et al., 2016; Mantel et al., 2012; Semerad et al., 2009). Thus, CITED2 controls multiple fundamental pathways in HSCs to safeguard their integrity.

DISCUSSION

Given that the functional role of CITED2 in HSC biology under unperturbed conditions remains poorly understood, here we conditionally deleted *Cited2* specifically from the hematopoietic system. While steady-state hematopoiesis in young and aging mice lacking *Cited2* was largely unaffected, HSCs were significantly depleted in young mice, at least in part via increased apoptosis, and failed to expand upon physiological aging. Importantly, these findings imply that HSCs under steady-state conditions require *Cited2* to regulate the size of their pool but not to sustain long-term multilineage hematopoiesis. Furthermore, while *Cited2*-deficient HSCs successfully homed to the BM upon transplantation, they completely failed to reconstitute hematopoiesis, indicating that HSCs critically require *Cited2* to function under stressful conditions imposed by transplantation. Finally, significantly, phenotypes elicited by *Cited2* deficiency demonstrate that unperturbed multilineage hematopoiesis can be sustained long term while the HSC pool is drastically depleted, thus underscoring the remarkable plasticity of the hematopoietic system under steady-state conditions.

Previous studies using poly(I:C)-inducible *Mx1-Cre* concluded that CITED2 regulates HSC survival and quiescence by repressing INK4A/ARF and HIF-1alpha, respectively (Du et al., 2012; Kranc et al., 2009). However, cellular and molecular mechanisms via which CITED2 controls the HSC pool under steady-state conditions and HSC reconstitution potential upon transplantation have remained largely unexplored. Here, we found that, under steady-state conditions, *Cited2*-deficient HSCs maintain their quiescent state but undergo increased apoptosis. Notably, *Cited2* is required for the expression of HSC survival and self-renewal regulator *Mcl1* (Campbell et al., 2010b; Opferman et al., 2005), whose hematopoiesis-specific overexpression partially rescued depletion of *Cited2*-deficient HSCs, and their ability to reconstitute long-term multilineage hematopoiesis. We therefore propose that the CITED2→MCL-1 axis protects the integrity of the HSC pool by promoting HSC survival under the steady-state conditions and upon transplantation. Furthermore, *Cited2* was necessary for normal expression of *Pten*, whose deletion results in a cell-autonomous HSC depletion and defective reconstitution potential (Yilmaz et al., 2006). Consistent with *Pten* downregulation, *Cited2*-deficient HSCs displayed increased signatures of PI3K/AKT and mTOR signaling, whose suppression by PTEN is essential for HSC maintenance (Lee et al., 2010; Yilmaz et al., 2006). These data are in concordance with the previous demonstration that *Cited2* deletion in HSCs leads to an increased AKT activity (Du et al., 2014). Indeed, given that PTEN is a negative regulator of AKT, our results help to explain increased AKT activation upon *Cited2* loss. Given that *Cited2*^{Het};*Pten*^{Het} HSCs displayed normal multilineage reconstitution potential but poorly contributed to the HSC pool upon transplantation, and that rapamycin did not rescue defects resulting from *Cited2* deficiency, it is likely that the CITED2→PTEN/PI3K/AKT/mTOR axis contributes to long-term HSC maintenance but is not solely critical for HSC functions.

Poly(I:C)-inducible *Mx1-Cre* deletion of *Cited2* results in loss of quiescence of HSCs, a phenotype that is partially mediated by HIF-1alpha (Du et al., 2012, 2014). Notably, however, our data indicate that *Vav-iCre*-mediated deletion of *Cited2* under homeostasis has no impact on HSC quiescence. Furthermore, our GSEA analyses showed that the hypoxia signature was in fact downregulated in *Cited2*-deficient HSCs. The discrepancies between our findings and

those by Du et al. (2012, 2014) may be explained by different strategies of gene deletion, as poly(I:C) (used to induce *Mx1-Cre*-driven gene ablation) is known to transiently alter HSCs (i.e., induce cycling, increase frequency, and alter phenotype), unlike *Vav-iCre*, which is constitutively expressed, and as such more accurately allows for gene deletion under steady-state conditions. Thus, it is possible that concurrent poly(I:C) administration and *Cited2* deletion in the *Mx1-Cre*-mediated model may exhibit exacerbated phenotypes not seen in the *Vav-iCre* model.

While in this study we focused on functional interrogation of the CITED2→MCL-1 and CITED2→PTEN axes, our work suggests that CITED2 is also likely to control other diverse pathways to coordinate HSC biology. Our analyses indicate that CITED2 represses multiple pathways downstream of c-MYC, E2F, and RUNX1 transcription factors and K-RAS and proinflammatory signaling pathways, whose upregulation has detrimental consequences for HSC integrity (Ichikawa et al., 2008; Kim et al., 2017; Pietras, 2017; Sasine et al., 2018; Wilson et al., 2004; Yilmaz et al., 2006). Finally, CITED2 is necessary for the expression of genes regulated by STAT3, STAT4, E2A, and ZFX transcription factors, which are essential for HSC maintenance (Galan-Caridad et al., 2007; Holmfeldt et al., 2016; Mantel et al., 2012; Semerad et al., 2009). Thus, given its dual action in gene transcription, we propose that CITED2 functions at the center of the transcriptional regulatory network to repress pathways detrimental to HSC integrity, and promote those necessary for HSC maintenance.

EXPERIMENTAL PROCEDURES

Mice

All mice were on the C57BL/6J genetic background. *Cited2*$^{fl/fl}$ (Kranc et al., 2009; MacDonald et al., 2008), *VavP-Mcl1* (Campbell et al., 2010b), *Pten*$^{fl/fl}$ (Yilmaz et al., 2006), and *Vav-iCre* (de Boer et al., 2003) mice were described previously. All transgenic and knockout mice were CD45.2$^+$. Recipient mice were CD45.1$^+$/CD45.2$^+$. All experiments on animals were performed under UK Home Office authorization.

Flow cytometry

BM, spleen, and PB samples were stained and analyzed as described previously (Guitart et al., 2017). FACS analyses were performed using an LSRFortessa (BD). Cell sorting was performed on a FACSAria Fusion (BD). Data were analyzed using FlowJo.

A combination of anti-mouse antibodies purchased from BD Biosciences, BioLegend, and Life Technologies was used. The following BD Biosciences antibodies were used: Fc block (cat. no. 553142), anti-CD4; biotin conjugated (cat. no. 553649), anti-CD5; biotin conjugated (cat. no. 553019), anti-CD8a; biotin conjugated (cat. no. 553029), anti-CD11b; biotin conjugated (cat. no. 553309), anti-CD45R/B220; biotin conjugated (cat. no. 553086), anti-Ter119; biotin conjugated (cat. no. 553672), anti-Gr-1/Ly-6G/C; biotin conjugated (cat. no. 553125), anti-CD34; FITC conjugated (cat. no. 553733), and streptavidin; BV421 conjugated (cat. no. 563259). BioLegend antibodies used were anti-c-Kit/CD117; APC conjugated (cat. no. 105812), anti-c-Kit/CD117; APC-Cy7 conjugated (cat. no. 105826), anti-Sca-1; APC-Cy7 conjugated (cat. no. 122520), anti-Sca-1; PB conjugated (cat. no. 108125), anti-CD48; PE conjugated (cat. no. 103406), anti-CD150; PE-Cy7 conjugated (cat. no. 115914), anti-CD135; APC conjugated (cat. no. 135310), anti-CD135; PE conjugated (cat. no. 135305), anti-CD16/32; APC-Cy7 conjugated (cat. no. 101328), anti-CD41; APC conjugated (cat. no. 133914), anti-CD105; PE conjugated (cat. no. 120408), anti-CD127; BV421 conjugated (cat. no. 135023), anti-TER-119; FITC conjugated (cat. no. 116206), streptavidin; PerCp conjugated (cat. no. 405213), anti-CD19; APC-Cy7 conjugated (cat. no. 115530), anti-CD45R/B220; APCCy7 conjugated (cat. no. 103224), anti-CD11b; APC conjugated (cat. no. 101211), anti-Gr-1/Ly-6G/C; PE-Cy7 conjugated (cat. no. 108416), anti-CD4; PE conjugated (cat. no. 130310), anti-CD8a; PE conjugated (cat. no. 100708), anti-CD45.1; FITC conjugated (cat. no. 110706), anti-CD45.2; PB conjugated (cat. no. 109820), anti-Ki67; FITC conjugated (cat. no. 652410). Annexin V; FITC conjugated (cat. no. 640906), and 7-AAD (cat. no. 420403) were purchased from BioLegend. DAPI was purchased from Life Technologies (cat. no. D1306).

Administration of NAC

Mice received 30 mg/mL of NAC (Sigma) in drinking water for 4 weeks. The water bottles containing NAC were changed twice a week.

ROS detection

For detection of mitochondrial super oxide, 3×10^6 BM cells stained first for LSK were resuspended in X-Vivo 15 medium (without phenol red) supplemented with 10% FCS, loaded with 5 μM MitoSox red (Invitrogen) for 20 min at 37°C and analyzed using FACS.

CFC assays

CFC assays were performed using MethoCult M3434 (STEMCELL Technologies). Colonies were tallied at day 10. For CFC replating, CFC1 cells were washed with IMDM then seeded in M3434.

Transplantation assays

Lethal irradiation of CD45.1$^+$/CD45.2$^+$ recipient mice was achieved using a split dose of 11 Gy (two doses of 5.5 Gy administered 4 h apart) at an average rate of 0.58 Gy/min using a Cesium 137 GammaCell 40 irradiator. For transplantations 100 HSCs or 2,000 LSK sorted from BM of 8- to 10-week old adult mice mixed with 200,000 support CD45.1$^+$ wild-type BM cells were injected into lethally irradiated CD45.1$^+$/CD45.2$^+$-recipient mice.

Homing assay

LSK cells sorted from CD45.2$^+$ BM were injected into CD45.1$^+$ lethally irradiated recipients (160,000 cells per mouse). After 18 h, recipients were sacrificed and BM CD45.2$^+$ chimerism was analyzed by FACS.

qRT-PCR

Gene expression analyses were performed as described previously (Guitart et al., 2017). Differences in input cDNA were normalized with *Actb* expression.

Statistical analyses

Statistical analyses were performed using GraphPad Prism software. p values were calculated using a Mann-Whitney U test.

RNA-seq, GSEA, and DNA motif analysis

RNA-seq was performed on sorted HSCs from *Cited2*^{CKO} and *Cited2*^{CTL} animals (n = 5 per genotype). On average, 6,200 cells per sample were collected and 53.7 million single-ended 85-bp reads per sample were sequenced. Reads were aligned to the GRCm38 mouse genome using HISAT2 v.2.1.0 (Kim et al., 2015), and read counts were assessed per gene using the Rsubread package v.2.0.1 in R. Differential expression analysis was further performed using the Wald test in DESeq2 v.1.24, and genes were ranked according to moderated t statistics computed by DESeq2 for GSEA. The ranked gene list was compared with gene lists in the hallmark subset of the MSigDb database v.7.0 using the GSEA software tool v.3.0 (Subramanian et al., 2005).

For DNA motif analysis of proximal promoter regions, lists of up- and downregulated genes (FDR < 0.05, fold change $> 20\%$) were mapped to gene loci using the basic set of ENCODE genomic annotation from Ensembl v.91. Next, proximal promoters were defined as the region from 200 bp upstream to 100 bp downstream of the transcription start site. Proximal promoter coordinates were shuffled within chromosomes using the bedtools shuffle tool with the –chrom flag to generate control DNA regions for motif analysis. Finally, DNA motifs overrepresented in promoters compared with control regions were identified by Homer v.4.10.

DATA AND CODE AVAILABILITY

The RNA-seq data are deposited in NCBI's Gene Expression Omnibus (accession no. GSE175372).

SUPPLEMENTAL INFORMATION

Supplemental information can be found online at https://doi.org/10.1016/j.stemcr.2021.10.001.

AUTHOR CONTRIBUTIONS

K.R.K., A.V.G., and H.L. designed the experiments and wrote the paper. A.V.G. and H.L. performed *in vivo* experiments, and data analyses and interpretation. L.N.L. and M.B. analyzed gene expression. K.J.C. and S.C. produced *VavP-Mcl1* transgenic mice. A.T., J.D., A.V., A.B., C.M., E.G., C.M.-C., C.S., L.A., and J.C. helped with *in vivo* experiments, FACS, and data analyses. D.O., B.G., and N.P.R. provided significant expertise to this study. K.R.K. and A.V.G. contributed equally to this work.

CONFLICTS OF INTERESTS

The authors declare no competing interests.

ACKNOWLEDGMENTS

This research was funded by Cancer Research UK (CRUK) Senior Cancer Research Fellowship and a CRUK Program Grant awarded to K.R.K. (C29967/A14633 and C29967/A26787). K.R.K.'s laboratory is also supported by grants from The Barts Charity, The Kay Kendall Leukaemia Fund and Blood Cancer UK. A.V.G. is supported by Ligue contre le cancer. This research was also funded in part by the Wellcome (203151/Z/16/Z) and the Medical Research Council (MC_PC_17230). We thank Vladimir Benes and Jelena Pistolic (Genomics Core Facility, EMBL, Heidelberg) for performing RNA-seq.

Received: June 2, 2021
Revised: October 1, 2021
Accepted: October 1, 2021
Published: October 28, 2021

REFERENCES

Andina, N.D., Sarangdhar, M., Tardivel, A., Bombaci, G., Hallal, M., Keller, I., and Allam, R. (2019). Higher vertebrate specific gene ribonuclease inhibitor (RNH1) is essential for adult hematopoietic stem cell function and cell cycle regulation. Blood *134*, 273.

Bamforth, S.D., Braganca, J., Eloranta, J.J., Murdoch, J.N., Marques, F.I., Kranc, K.R., Farza, H., Henderson, D.J., Hurst, H.C., and Bhattacharya, S. (2001). Cardiac malformations, adrenal agenesis, neural crest defects and exencephaly in mice lacking Cited2, a new Tfap2 co-activator. Nat. Genet. *29*, 469–474.

Bamforth, S.D., Braganca, J., Farthing, C.R., Schneider, J.E., Broadbent, C., Michell, A.C., Clarke, K., Neubauer, S., Norris, D., Brown, N.A., et al. (2004). Cited2 controls left-right patterning and heart development through a Nodal-Pitx2c pathway. Nat. Genet. *36*, 1189–1196.

Bhattacharya, S., Michels, C.L., Leung, M.K., Arany, Z.P., Kung, A.L., and Livingston, D.M. (1999). Functional role of p35srj, a novel p300/CBP binding protein, during transactivation by HIF-1. Genes Dev. *13*, 64–75.

Campbell, C.J., Lee, J.B., Levadoux-Martin, M., Wynder, T., Xenocostas, A., Leber, B., and Bhatia, M. (2010a). The human stem cell hierarchy is defined by a functional dependence on Mcl-1 for self-renewal capacity. Blood *116*, 1433–1442.

Campbell, K.J., Bath, M.L., Turner, M.L., Vandenberg, C.J., Bouillet, P., Metcalf, D., Scott, C.L., and Cory, S. (2010b). Elevated Mcl-1 perturbs lymphopoiesis, promotes transformation of hematopoietic stem/progenitor cells, and enhances drug resistance. Blood *116*, 3197–3207.

Chen, Y., Haviernik, P., Bunting, K.D., and Yang, Y.C. (2007). Cited2 is required for normal hematopoiesis in the murine fetal liver. Blood *110*, 2889–2898.

Chou, Y.T., Hsieh, C.H., Chiou, S.H., Hsu, C.F., Kao, Y.R., Lee, C.C., Chung, C.H., Wang, Y.H., Hsu, H.S., Pang, S.T., et al. (2012). CITED2 functions as a molecular switch of cytokine-induced proliferation and quiescence. Cell Death Differ *19*, 2015–2028.

Chou, Y.T., and Yang, Y.C. (2006). Post-transcriptional control of Cited2 by transforming growth factor beta. Regulation via Smads and Cited2 coding region. J. Biol. Chem. *281*, 18451–18462.

Dahlin, J.S., Hamey, F.K., Pijuan-Sala, B., Shepherd, M., Lau, W.W.Y., Nestorowa, S., Weinreb, C., Wolock, S., Hannah, R., Diamanti, E., et al. (2018). A single-cell hematopoietic landscape resolves 8 lineage trajectories and defects in Kit mutant mice. Blood *131*, e1–e11.

de Boer, J., Williams, A., Skavdis, G., Harker, N., Coles, M., Tolaini, M., Norton, T., Williams, K., Roderick, K., Potocnik, A.J., and Kioussis, D. (2003). Transgenic mice with hematopoietic and lymphoid specific expression of Cre. Eur. J. Immunol. *33*, 314–325.

Du, J., Chen, Y., Li, Q., Han, X., Cheng, C., Wang, Z., Danielpour, D., Dunwoodie, S.L., Bunting, K.D., and Yang, Y.C. (2012). HIF-1alpha deletion partially rescues defects of hematopoietic stem cell quiescence caused by Cited2 deficiency. Blood *119*, 2789–2798.

Du, J., Li, Q., Tang, F., Puchowitz, M.A., Fujioka, H., Dunwoodie, S.L., Danielpour, D., and Yang, Y.C. (2014). Cited2 is required for the maintenance of glycolytic metabolism in adult hematopoietic stem cells. Stem Cells Dev *23*, 83–94.

Fernandes, M.T., Calado, S.M., Mendes-Silva, L., and Braganca, J. (2020). CITED2 and the modulation of the hypoxic response in cancer. World J. Clin. Oncol. *11*, 260–274.

Galan-Caridad, J.M., Harel, S., Arenzana, T.L., Hou, Z.E., Doetsch, F.K., Mirny, L.A., and Reizis, B. (2007). Zfx controls the self-renewal of embryonic and hematopoietic stem cells. Cell *129*, 345–357.

Geiger, H., de Haan, G., and Florian, M.C. (2013). The ageing haematopoietic stem cell compartment. Nat. Rev. Immunol. *13*, 376–389.

Gudmundsson, K.O., Nguyen, N., Oakley, K., Han, Y., Gudmundsdottir, B., Liu, P., Tessarollo, L., Jenkins, N.A., Copeland, N.G., and Du, Y. (2020). Prdm16 is a critical regulator of adult long-term hematopoietic stem cell quiescence. Proc. Natl. Acad. Sci. U S A *117*, 31945–31953.

Guertin, D.A., and Sabatini, D.M. (2007). Defining the role of mTOR in cancer. Cancer Cell *12*, 9–22.

Guitart, A.V., Panagopoulou, T.I., Villacreces, A., Vukovic, M., Sepulveda, C., Allen, L., Carter, R.N., van de Lagemaat, L.N., Morgan, M., Giles, P., et al. (2017). Fumarate hydratase is a critical metabolic regulator of hematopoietic stem cell functions. J. Exp. Med. *214*, 719–735.

Holmfeldt, P., Ganuza, M., Marathe, H., He, B., Hall, T., Kang, G., Moen, J., Pardieck, J., Saulsberry, A.C., Cico, A., et al. (2016). Functional screen identifies regulators of murine hematopoietic stem cell repopulation. J. Exp. Med. *213*, 433–449.

Ichikawa, M., Goyama, S., Asai, T., Kawazu, M., Nakagawa, M., Takeshita, M., Chiba, S., Ogawa, S., and Kurokawa, M. (2008). AML1/Runx1 negatively regulates quiescent hematopoietic stem cells in adult hematopoiesis. J. Immunol. *180*, 4402–4408.

Ivanova, N.B., Dimos, J.T., Schaniel, C., Hackney, J.A., Moore, K.A., and Lemischka, I.R. (2002). A stem cell molecular signature. Science *298*, 601–604.

Juntilla, M.M., Patil, V.D., Calamito, M., Joshi, R.P., Birnbaum, M.J., and Koretzky, G.A. (2010). AKT1 and AKT2 maintain hematopoietic stem cell function by regulating reactive oxygen species. Blood *115*, 4030–4038.

Kim, D., Langmead, B., and Salzberg, S.L. (2015). HISAT: a fast spliced aligner with low memory requirements. Nat. Methods *12*, 357–360.

Kim, E., Cheng, Y., Bolton-Gillespie, E., Cai, X., Ma, C., Tarangelo, A., Le, L., Jambhekar, M., Raman, P., Hayer, K.E., et al. (2017). Rb family proteins enforce the homeostasis of quiescent hematopoietic stem cells by repressing Socs3 expression. J. Exp. Med. *214*, 1901–1912.

Kim, G.D., Das, R., Rao, X., Zhong, J., Deiuliis, J.A., Ramirez-Bergeron, D.L., Rajagopalan, S., and Mahabeleshwar, G.H. (2018). CITED2 restrains proinflammatory macrophage activation and response. Mol. Cell Biol *38*, e00452–17.

Korthuis, P.M., Berger, G., Bakker, B., Rozenveld-Geugien, M., Jaques, J., de Haan, G., Schuringa, J.J., Vellenga, E., and Schepers, H. (2015). CITED2-mediated human hematopoietic stem cell maintenance is critical for acute myeloid leukemia. Leukemia *29*, 625–635.

Kranc, K.R., Bamforth, S.D., Braganca, J., Norbury, C., van Lohuizen, M., and Bhattacharya, S. (2003). Transcriptional coactivator Cited2 induces Bmi1 and Mel18 and controls fibroblast proliferation via Ink4a/ARF. Mol. Cell Biol. *23*, 7658–7666.

Kranc, K.R., Oliveira, D.V., Armesilla-Diaz, A., Pacheco-Leyva, I., Catarina Matias, A., Luisa Escapa, A., Subramani, C., Wheadon, H., Trindade, M., Nichols, J., et al. (2015). Acute loss of Cited2 impairs Nanog expression and decreases self-renewal of mouse embryonic stem cells. Stem Cells *33*, 699–712.

Kranc, K.R., Schepers, H., Rodrigues, N.P., Bamforth, S., Villadsen, E., Ferry, H., Bouriez-Jones, T., Sigvardsson, M., Bhattacharya, S., Jacobsen, S.E., and Enver, T. (2009). Cited2 is an essential regulator of adult hematopoietic stem cells. Cell Stem Cell *5*, 659–665.

Lawson, H., Sepulveda, C., van de Lagemaat, L.N., Durko, J., Barile, M., Tavosanis, A., Georges, E., Shmakova, A., Timms, P., Carter, R.N., et al. (2021). JMJD6 promotes self-renewal and regenerative capacity of hematopoietic stem cells. Blood Adv. *5*, 889–899.

Lee, J.Y., Nakada, D., Yilmaz, O.H., Tothova, Z., Joseph, N.M., Lim, M.S., Gilliland, D.G., and Morrison, S.J. (2010). mTOR activation induces tumor suppressors that inhibit leukemogenesis and deplete hematopoietic stem cells after Pten deletion. Cell Stem Cell *7*, 593–605.

Lee, J.Y., Taub, P.J., Wang, L., Clark, A., Zhu, L.L., Maharam, E.R., Leong, D.J., Ramcharan, M., Li, Z., Liu, Z., et al. (2009). Identification of CITED2 as a negative regulator of fracture healing. Biochem. Biophys. Res. Commun. *387*, 641–645.

Li, Q., Ramirez-Bergeron, D.L., Dunwoodie, S.L., and Yang, Y.C. (2012). Cited2 gene controls pluripotency and cardiomyocyte differentiation of murine embryonic stem cells through Oct4 gene. J. Biol. Chem. *287*, 29088–29100.

Liu, L., He, Z., Xu, L., Lu, L., Feng, H., Leong, D.J., Kim, S.J., Hirsh, D.M., Majeska, R.J., Goldring, M.B., et al. (2019). CITED2 mediates the mechanical loading-induced suppression of adipokines in the infrapatellar fat pad. Ann. N. Y Acad. Sci. *1442*, 153–164.

Liu, Y., Elf, S.E., Miyata, Y., Sashida, G., Liu, Y., Huang, G., Di Giandomenico, S., Lee, J.M., Deblasio, A., Menendez, S., et al. (2009). p53 regulates hematopoietic stem cell quiescence. Cell Stem Cell *4*, 37–48.

MacDonald, S.T., Bamforth, S.D., Chen, C.M., Farthing, C.R., Franklyn, A., Broadbent, C., Schneider, J.E., Saga, Y., Lewandoski, M., and Bhattacharya, S. (2008). Epiblastic Cited2 deficiency results in cardiac phenotypic heterogeneity and provides a mechanism for haploinsufficiency. Cardiovasc. Res. *79*, 448–457.

Magee, J.A., Ikenoue, T., Nakada, D., Lee, J.Y., Guan, K.L., and Morrison, S.J. (2012). Temporal changes in PTEN and mTORC2 regulation of hematopoietic stem cell self-renewal and leukemia suppression. Cell Stem Cell *11*, 415–428.

Maillard, I., Chen, Y.X., Friedman, A., Yang, Y., Tubbs, A.T., Shestova, O., Pear, W.S., and Hua, X. (2009). Menin regulates the function of hematopoietic stem cells and lymphoid progenitors. Blood *113*, 1661–1669.

Mantel, C., Messina-Graham, S., Moh, A., Cooper, S., Hangoc, G., Fu, X.Y., and Broxmeyer, H.E. (2012). Mouse hematopoietic cell-targeted STAT3 deletion: stem/progenitor cell defects, mitochondrial dysfunction, ROS overproduction, and a rapid aging-like phenotype. Blood *120*, 2589–2599.

McCracken, M.N., George, B.M., Kao, K.S., Marjon, K.D., Raveh, T., and Weissman, I.L. (2016). Normal and neoplastic stem cells. Cold Spring Harb Symp. Quant Biol. *81*, 1–9.

Menendez-Gonzalez, J.B., Vukovic, M., Abdelfattah, A., Saleh, L., Almotiri, A., Thomas, L.A., Agirre-Lizaso, A., Azevedo, A., Menezes, A.C., Tornillo, G., et al. (2019). Gata2 as a crucial regulator of stem cells in adult hematopoiesis and acute myeloid leukemia. Stem Cell Rep. *13*, 291–306.

Nestorowa, S., Hamey, F.K., Pijuan Sala, B., Diamanti, E., Shepherd, M., Laurenti, E., Wilson, N.K., Kent, D.G., and Gottgens, B. (2016). A single-cell resolution map of mouse hematopoietic stem and progenitor cell differentiation. Blood *128*, e20–31.

Opferman, J.T., Iwasaki, H., Ong, C.C., Suh, H., Mizuno, S., Akashi, K., and Korsmeyer, S.J. (2005). Obligate role of anti-apoptotic MCL-1 in the survival of hematopoietic stem cells. Science *307*, 1101–1104.

Pietras, E.M. (2017). Inflammation: a key regulator of hematopoietic stem cell fate in health and disease. Blood *130*, 1693–1698.

Rossi, L., Lin, K.K., Boles, N.C., Yang, L., King, K.Y., Jeong, M., Mayle, A., and Goodell, M.A. (2012). Less is more: unveiling the functional core of hematopoietic stem cells through knockout mice. Cell Stem Cell *11*, 302–317.

Sasine, J.P., Himburg, H.A., Termini, C.M., Roos, M., Tran, E., Zhao, L., Kan, J., Li, M., Zhang, Y., de Barros, S.C., et al. (2018). Wild-type Kras expands and exhausts hematopoietic stem cells. JCI Insight *3*, e98197.

Semerad, C.L., Mercer, E.M., Inlay, M.A., Weissman, I.L., and Murre, C. (2009). E2A proteins maintain the hematopoietic stem cell pool and promote the maturation of myelolymphoid and myeloerythroid progenitors. Proc. Natl. Acad. Sci. U S A *106*, 1930–1935.

Sender, R., and Milo, R. (2021). The distribution of cellular turnover in the human body. Nat. Med. *27*, 45–48.

Spencer, J.A., Ferraro, F., Roussakis, E., Klein, A., Wu, J., Runnels, J.M., Zaher, W., Mortensen, L.J., Alt, C., Turcotte, R., et al. (2014). Direct measurement of local oxygen concentration in the bone marrow of live animals. Nature *508*, 269–273.

Subramanian, A., Tamayo, P., Mootha, V.K., Mukherjee, S., Ebert, B.L., Gillette, M.A., Paulovich, A., Pomeroy, S.L., Golub, T.R., Lander, E.S., and Mesirov, J.P. (2005). Gene set enrichment analysis: a knowledge-based approach for interpreting genome-wide expression profiles. Proc. Natl. Acad. Sci. U S A *102*, 15545–15550.

Tien, E.S., Davis, J.W., and Vanden Heuvel, J.P. (2004). Identification of the CREB-binding protein/p300-interacting protein CITED2 as a peroxisome proliferator-activated receptor alpha coregulator. J. Biol. Chem. *279*, 24053–24063.

Vidal, M., Cusick, M.E., and Barabasi, A.L. (2011). Interactome networks and human disease. Cell *144*, 986–998.

Wang, Y.H., Israelsen, W.J., Lee, D., Yu, V.W., Jeanson, N.T., Clish, C.B., Cantley, L.C., Vander Heiden, M.G., and Scadden, D.T. (2014). Cell-state-specific metabolic dependency in hematopoiesis and leukemogenesis. Cell *158*, 1309–1323.

Weninger, W.J., Floro, K.L., Bennett, M.B., Withington, S.L., Preis, J.I., Barbera, J.P., Mohun, T.J., and Dunwoodie, S.L. (2005). Cited2 is required both for heart morphogenesis and establishment of the left-right axis in mouse development. Development *132*, 1337–1348.

Wilson, A., Murphy, M.J., Oskarsson, T., Kaloulis, K., Bettess, M.D., Oser, G.M., Pasche, A.C., Knabenhans, C., Macdonald, H.R., and Trumpp, A. (2004). c-Myc controls the balance between hematopoietic stem cell self-renewal and differentiation. Genes Dev. *18*, 2747–2763.

Withington, S.L., Scott, A.N., Saunders, D.N., Lopes Floro, K., Preis, J.I., Michalicek, J., Maclean, K., Sparrow, D.B., Barbera, J.P., and Dunwoodie, S.L. (2006). Loss of Cited2 affects trophoblast formation and vascularization of the mouse placenta. Dev. Biol. *294*, 67–82.

Yilmaz, O.H., Valdez, R., Theisen, B.K., Guo, W., Ferguson, D.O., Wu, H., and Morrison, S.J. (2006). Pten dependence distinguishes haematopoietic stem cells from leukaemia-initiating cells. Nature *441*, 475–482.

Yin, Z., Haynie, J., Yang, X., Han, B., Kiatchoosakun, S., Restivo, J., Yuan, S., Prabhakar, N.R., Herrup, K., Conlon, R.A., et al. (2002). The essential role of Cited2, a negative regulator for HIF-1alpha, in heart development and neurulation. Proc. Natl. Acad. Sci. U S A *99*, 10488–10493.

Zhang, J., Grindley, J.C., Yin, T., Jayasinghe, S., He, X.C., Ross, J.T., Haug, J.S., Rupp, D., Porter-Westpfahl, K.S., Wiedemann, L.M., et al. (2006). PTEN maintains haematopoietic stem cells and acts in lineage choice and leukaemia prevention. Nature *441*, 518–522.

Stem Cell Reports, Volume *16*

Supplemental Information

CITED2 coordinates key hematopoietic regulatory pathways to maintain the HSC pool in both steady-state hematopoiesis and transplantation

Hannah Lawson, Louie N. van de Lagemaat, Melania Barile, Andrea Tavosanis, Jozef Durko, Arnaud Villacreces, Aarushi Bellani, Christopher Mapperley, Elise Georges, Catarina Martins-Costa, Catarina Sepulveda, Lewis Allen, Joana Campos, Kirsteen J. Campbell, Dónal O'Carroll, Berthold Göttgens, Suzanne Cory, Neil P. Rodrigues, Amelie V. Guitart, and Kamil R. Kranc

Supplemental Figures

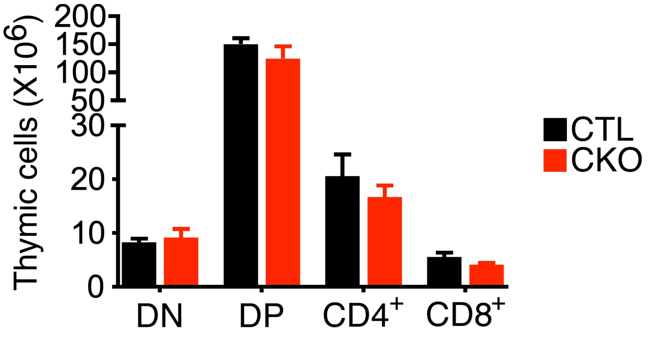

Figure S1. Haematopoiesis-specific *Cited2* deletion has no impact on T cell development. Total number of double-negative (DN; $CD4^-CD8^-$), double-positive (DP; $CD4^+CD8^+$), $CD4^+$ and $CD8^+$ T cells in the thymi. Data are mean ± SEM (n=6-9).

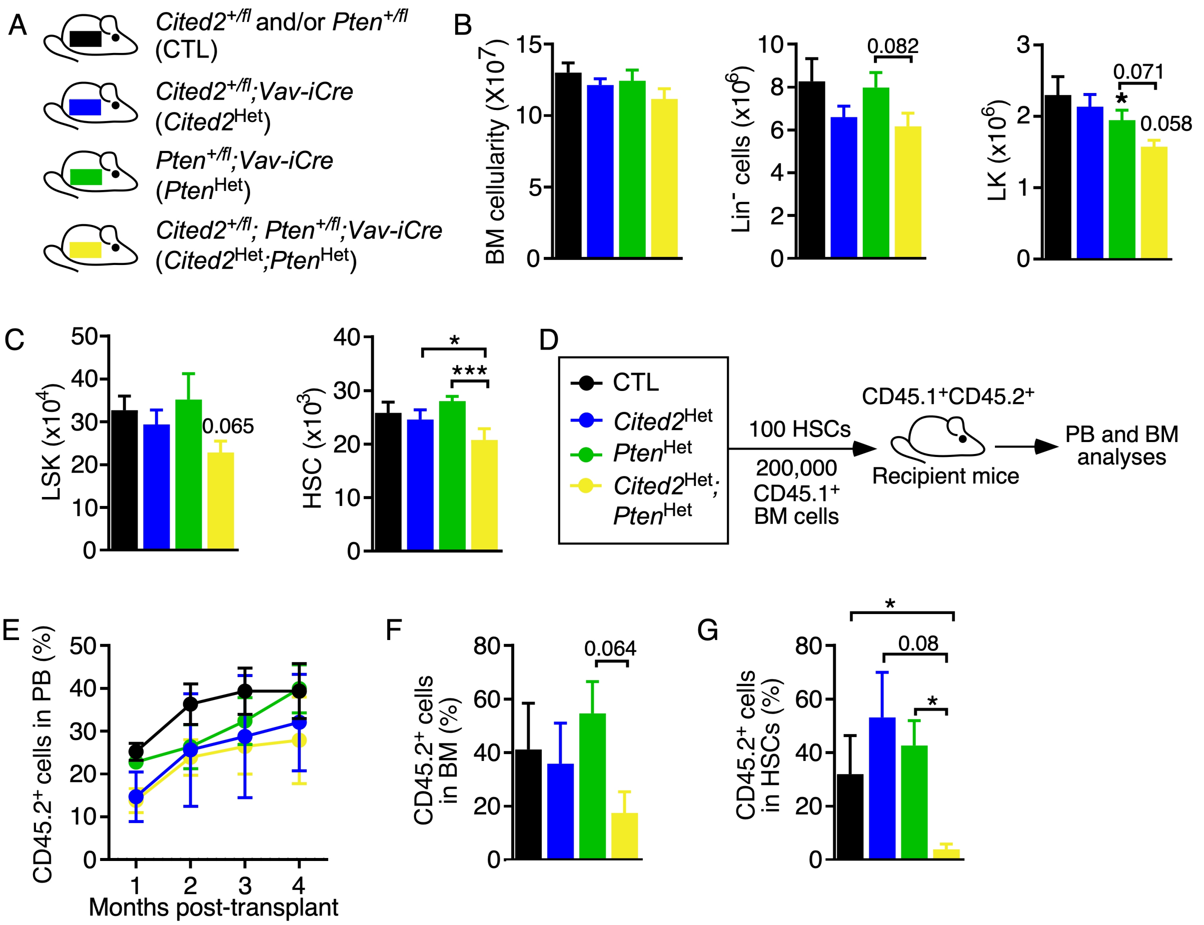

Figure S2. Deletion of one allele of *Pten* impairs maintenance of HSCs lacking one allele of *Cited2*. (**A**) Schematic representation of experimental mouse cohorts; *Cited2*$^{+/fl}$ or *Pten*$^{+/fl}$ (CTL), *Cited2*$^{+/fl}$*;Vav-iCre* (*Cited2*^{HET}), *Pten*$^{+/fl}$*;Vav-iCre* (*Pten*^{HET}) and *Cited2*$^{+/fl}$;*Pten*$^{+/fl}$*;Vav-iCre* (*Cited2*^{HET};*Pten*^{HET}). (**B**) BM cellularity and total numbers of Lin^{-} cells, LK cells in BM of 8 to 10-week-old mice (n=6-8). Data are mean ± SEM. (**C**) Total numbers of LSK cells and HSCs in BM of 8 to 10-week-old mice (n=6-8). Data are mean ± SEM.(**D**) Transplantation assay: 100 HSCs from CTL, *Cited2*^{HET}, (*Pten*^{HET}) and *Cited2*^{HET}*;Pten*^{HET} were transplanted into lethally irradiated recipients together with 2x10^{5} support CD45.1^{+} total BM cells. (**E**) Percentage of donor-derived CD45.2^{+} cells in PB following transplantation (n=4 recipients per donor; number of donors: CTL n=3, *Cited2*^{HET} n=3, *Pten*^{HET} n=3, *Cited2*^{HET};*Pten*^{HET} n=3). Data are mean ± SEM. (**F**) Percentage of donor-derived CD45.2^{+} cells in total BM compartment of the recipient mice 16 weeks after transplantation. Data are mean ± SEM. (**G**) Percentage of donor-derived CD45.2^{+} cells in the BM HSC compartment. Data are mean ± SEM. *, P<0.05; ***, P<0.001.

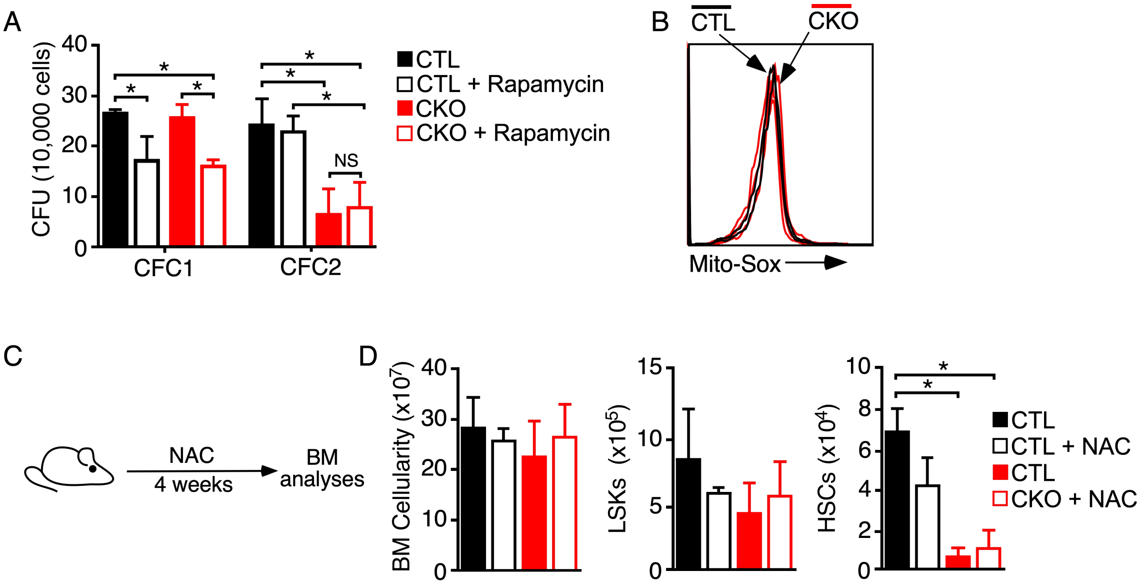

Figure S3. Rapamycin or N-acetyl-L-cysteine (NAC) do not rescue defects resulting from *Cited2* deficiency. (A) BM cells from 8- to 10-week-old *Cited2*^{CKO} and *Cited2*^{CTL} mice (n=4 per genotype) were placed in colony forming cell (CFC) assays in the presence and absence of 1μM rapamycin. CFC1 colonies were counted 10 days after plating, and re-plated into CFC2. CFC2 colonies were counted after 10 days in culture. Data are mean ± SEM. *, P < 0.05 (**B**) ROS levels in HSCs from *Cited2*^{CKO} and *Cited2*^{CTL} mice measured by MitoSOX (mitochondrial superoxide–sensitive fluorophore). (**C**) Experimental design. 9-week-old mice received 30 mg/mL of NAC in drinking water for 4 weeks. After 4 weeks of NAC administration, BM was analysed by FACS. (**D**) The graphs show total BM cellularity and total numbers in BM LSK cells and HSCs of *Cited2*^{CKO} and *Cited2*^{CTL} mice (n=4-6). Data are mean ± SEM. *, P < 0.05

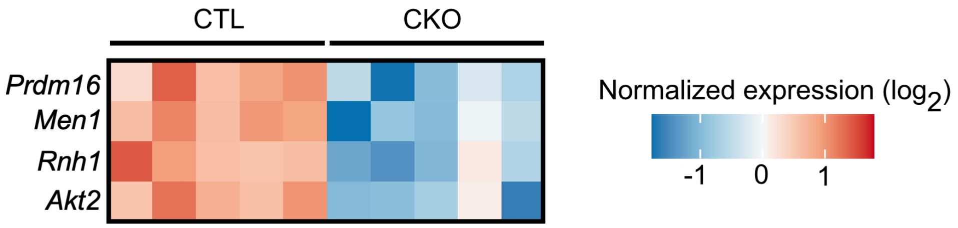

Figure S4. Decreased expression of *Prdm16*, *Men1*, *Rnh1* and *Akt2* in HSCs lacking *Cited2*. Heatmaps of significantly dysregulated genes (FDR < 0.05) essential for HSC functions in *Cited2*^{CKO} compared with *Cited2*^{CTL} HSCs (n=5).

Supplemental Experimental Procedures

Flow cytometry

Briefly, BM cells were obtained by crushing tibias and femurs with a pestle and mortar. Spleen cells were obtained by mashing the tissue through a 70μm strainer. Single cell suspensions from BM, spleen or PB were incubated in Fc block and then stained with antibodies. For HSC analyses, following incubation with Fc block, unfractionated BM cell suspensions were stained with lineage markers containing biotin-conjugated anti-CD4, anti-CD5, anti-CD8a, anti-CD11b, anti-B220, anti-Gr-1 and anti-Ter119 antibodies together with APC or APC/Cy7-conjugated anti-c-Kit, APC/Cy7- or PB-conjugated anti-Sca-1, PE-conjugated anti-CD48 and PE-Cy7-conjugated anti-CD150, and for some analysis APC-conjugated anti-CD135 and FITC-conjugated anti-CD34 antibodies were also added. For analysis of committed progenitor cells unfractionated BM cell suspensions were stained with lineage markers containing biotin-conjugated anti-CD4, anti-CD5, anti-CD8a, anti-CD11b, anti-B220 and anti-Gr-1 antibodies together with BV711-conjugated anti-c-Kit, APCCy7-conjugated CD16/32, APC-conjugated CD41, PE-conjugated CD105, BV421-conjugated CD127, PE-conjugated CD135 (Flt3), FITC-conjugated Ter119. Biotin-conjugated antibodies were then stained with Pacific Blue-conjugated or PerCP-conjugated streptavidin. To distinguish CD45.2^{+}-donor derived HSCs in recipient mice, FITC-conjugated anti-CD45.1 and Pacific Blue-conjugated anti-CD45.2 antibodies were included in the antibody cocktail described above. The multilineage reconstitution of recipient mice was determined by staining the BM or PB cell suspensions of the recipient mice with FITC-conjugated anti-CD45.1, Pacific Blue-conjugated anti-CD45.2, PE-conjugated anti-CD4 and -CD8a, PE/Cy7-conjugated anti-Gr-1, APC-conjugated anti-CD11b, APC-Cy7-conjugated anti-CD19 and anti-B220). For cell cycle analyses, cells were further stained with FITC-conjugated Ki67 and DAPI. For analyses of apoptosis, cells were further stained with FITC-conjugated Annexin-V and DAPI. In all analyses, 7-AAD or DAPI were used for dead cell exclusion. Flow cytometry analyses were performed using a LSRFortessa (BD). Cell sorting was performed on a FACSAria Fusion (BD). Analysis was done using FlowJo.
